# Food preferences of similarly raised and kept captive dogs and wolves

**DOI:** 10.1371/journal.pone.0203165

**Published:** 2018-09-20

**Authors:** Akshay Rao, Friederike Range, Kerstin Kadletz, Kurt Kotrschal, Sarah Marshall-Pescini

**Affiliations:** 1 Wolf Science Center, Konrad-Lorenz Institute of Ethology, University of Veterinary Medicine, Vienna, Austria; 2 Comparative Cognition, Messerli Research Institute, University of Veterinary Medicine, Vienna, Medical University of Vienna, University of Vienna, Vienna, Austria; 3 Department of Behavioural Biology, University of Vienna, Vienna, Austria; Memorial University of Newfoundland, CANADA

## Abstract

Food preferences may be driven by a species’ ecology. Closely related species such as dogs and wolves may have evolved preferences for different foods owing to their differing foraging styles. Wolves have been shown to be more persistent in problem-solving experiments and more risk-prone in a foraging task. A possible element affecting these (and other) results is a potential wolf-dog difference in food preferences. To address this possibility, we tested similarly raised and kept dogs and wolves in two different food choice tasks, a classic two-choice task and a multiple-choice paradigm. We predicted that if dogs have adapted to a more opportunistic, scavenging foraging style, they would show a weaker preference for meat over starch rich foods (such as kibble) and be less affected by hunger than wolves. Alternatively, given the recentness of the new niche dogs have created, we predicted no substantial differences between dogs’ and wolves’ food preferences. We found that our subjects did not differ in their preference for meat over kibble in either paradigm. However, wolves’ (but not dogs’) choice patterns were affected by satiation, with wolves being less “selective” when hungry. Furthermore, when fed before testing, wolves were more selective than dogs. These differences were more noticeable in the multiple-choice paradigm than the two-choice task, suggesting that the former, novel paradigm may be more sensitive and better capable of evaluating food preferences in a diverse range of species. Overall, we found that the distinct differences in wolves’ and dogs’ ecology and foraging styles do not appear to have affected their food preferences and thus, differences in food preferences are unlikely to have influenced results of previous experiments demonstrating wolf-dog differences in cognitive skills.

## Introduction

The evolution of food preferences may be driven by the habitat a species has evolved in, energetic and protein requirements, and resource distribution [1]. For example, the food preferences of captive spider monkeys were positively correlated with the foods’ energy content and negatively with its water content, a result in line with what would be expected from their frugivorous feeding niche and opportunistic feeding style [2]. Similarly, several predators (including domestic dogs, cats, mink and fish) have been shown to prefer protein rich foods [3–6] in accordance with their carnivorous feeding niches. Food preferences are the behavioural fingerprints of evolved feeding niches [7]. An example of this phenomenon was shown in a study on primates in Madagascar where several related species of Lemurs cohabiting a forest showed preferences for leaves with different chemical compositions [8]. Hence, food preferences, feeding niche (or dietary specializations) and foraging style (or strategies used to obtain food) [9] appear to be generally connected in several species [10,11].

Canines are a relevant taxon to study these connections, since several closely related canines have fundamentally different foraging styles; for example, domestic dogs and their closest living relatives, wolves [12]. These differences are most prominently noticeable in free-ranging dogs, which make up over 75% of the world’s dog population (reviewed in [13,14]). While dogs are capable of hunting [15], they are primarily solitary scavengers [16,17] that thrive around human settlements and feed predominantly and indiscriminately on human refuse [18]. Close analyses of free-ranging dogs’ diet have revealed that the largest components of their diet are grains and human faeces [16,17,19,20]. Wolves, on the other hand, while occasionally observed scavenging on human refuse, are specialized hunters [16] and often hunt in packs. Considering their variable and often low success rate (between 10% and 49% per chase), hunting is thought to require an extraordinary level of persistence and food-motivation [21]. The dependence on different food resources is also evident in their genes, with dogs showing better starch digestion than wolves [22] (but see [23]). Another crucial aspect to consider is the effect of hunger, which may affect preference patterns in both dogs and wolves. Hunger is a motivational factor [24,25], and may lead to animals consuming novel foods [26] and even modify their foraging styles [27]. The variation in dogs’ and wolves’ foraging styles could thus be due to motivational changes induced by hunger and may differently affected their preference for specific food types.

The different socio-ecologies of dogs and wolves is postulated to have shaped the way they approach both social and independent problem-solving tasks [17]. For instance, in line with wolves’ dependence on cooperation in both hunting and pup-rearing, wolves outperformed dogs in a cooperative string-pulling task [28] and showed more food sharing than dogs [29]. Furthermore, compared to dogs, wolves were more persistent in extractive tasks involving food [30–32] and took more risks in a foraging task, when the choice was between a safe lower quality food reward, and a less stable/riskier higher quality one [33]. However, considering the different feeding ecologies of dogs and wolves, one possible underlying motivation for wolves’ increased persistence, better problem-solving skills and more risk-taking behaviour is differences in dogs’ and wolves’ food preferences.

To address this possibility, in the current study we tested similarly raised and kept dogs and wolves in two different food choice tasks. We first used a classic two-choice paradigm, where subjects could choose one of two presented foods. This is a common paradigm for testing food preferences in several taxa [1,2,34–40] including dogs and wolves [33,41–43]. However, although widely used, this paradigm has some shortcomings [44]: while it tells us which food an animal prefers from a pair, it is difficult to say whether the animal would choose similarly when presented with multiple choices. Furthermore, task contingencies and experience with other, similar two-choice tasks may affect the animals’ behaviours (e.g. side biases in two-choice tasks). Using a second paradigm and assessing the consistency in the animals’ preferences between tests would provide better insight into the animals’ preferences. Hence, we also adopted a “cafeteria” paradigm where subjects could choose three out of five simultaneously presented food types.

Foraging styles may affect food preferences, and, as outlined above, wolves and dogs show some differentiation in their foraging styles (group hunting ungulates vs. scavenging of human refuse). Our main aim was to assess the hypothesis that dogs’ and wolves’ food preferences may have changed during domestication, potentially due to changes in their foraging styles. Specifically, considering that dogs adapted to a more opportunistic, scavenging style during domestication, and show genetic adaptions to starch, they may show a less strict preference for a single food type, and show a weaker preference for meat over starch-rich food. Based on this hypothesis, we predicted that compared to wolves, dogs (1) would show a weaker preference for meat over starch-rich food (i.e. dog kibble) in the two-choice task and (2) would be less likely to choose meat and chicks as their first choice in the cafeteria paradigm. We also predicted that dogs (3) would have more choice diversity than wolves (i.e., less preference than wolves for certain foods) in the cafeteria task, and (4) would choose nearby foods (foods that are in immediate proximity of a previously chosen food) regardless of the food type, while wolves, having a stronger preference for meat than dogs, would be more likely to choose nearby foods if they were meat or chicks.

Although the feeding niche of dogs and wolves has changed during the course of domestication, the new niche dogs produced is recent and there is a continuum in dogs’ and wolves’ foraging styles (wolves show scavenging behaviours [16,45] and populations of dogs are known to hunt small ungulates in groups [15,46,47]). The null hypothesis then, is that dogs’ feeding ecology has not affected their food preferences when compared to wolves, and dogs still prefer food high in energy and protein [6]. Based on this hypothesis no substantial differences in dogs’ and wolves’ food preference patterns would be expected.

Since preferences may be linked to the nutritive value of food [11], we conducted nutritional analyses of the food types used. Finally, since hunger may influence food preferences, we tested subjects in two different satiation states in both paradigms. We predicted that when hungry, subjects would spend more time trying to acquire inaccessible food (i.e., during “inspection” and at the end of a test trial when the apparatus is locked) (See “Testing Phase” under “Procedure” in the “Cafeteria Paradigm” section).

## Materials and methods

### Ethical approval

Special permission to use animals (wolves) in cognitive studies (such as this one) is not required in Austria (Tierversuchsgesetz 2012—TVG 2012). The Tierversuchskommission am Bundesministerium für Wissenschaft und Forschung (Austria) allows research without special permissions regarding animals. The ethical approval for this study was obtained from the ‘Ethik und Tierschutzcommission’ of the University of Veterinary Medicine (Protocol number ETK-10/03/2016). The Wolf Science Center is in the game park Ernstbrunn (License No.: AT00012014). The CITES permits for our animals are: 2008: Zoo Herberstein, Austria: AT08-B-0998, AT08-B-0996, AT08-B-0997; 2009: Zoo Basel, Switzerland: AT09-E-0061, Triple D Farm, USA: AT09-E-0018; 2010: Parc Safari, Canada: AT10-E-0018; 2012: Minnesota Wildlife Connection, USA: 12AT330200INEGCJ93, Haliburton Forest, Canada: AT12-E0020. The individuals appearing in the figures and videos in this manuscript have given written informed consent (as outlined in PLOS consent form) to publish these media.

### Subjects

A total of 14 wolves (6 F, 8 M) and 19 medium sized, mixed-breed dogs (7 F, 12 M), similarly raised and kept in conspecific packs at the Wolf Science Center, Ernstbrunn Wild Park, Austria, participated in the entire study. Eleven wolves (5 F, 7 M; mean age 3.5 years, SD 1.7 years) and 10 dogs (4 F, 6 M; mean age 3 years, SD 0.6 years) participated in the two-choice task and 12 wolves (4 F, 8 M; mean age 6.3 years, SD 1.7 years) and 17 dogs (6 F, 10 M; mean age 4 years, SD 1.6 years), participated in the cafeteria paradigm (Table 1).

**Table 1:** Details of the subjects that participated in each testing paradigm.

Subject	Group	Sex	Date of Birth	Age when tested	
				Two-Choice Task	Cafeteria Paradigm
Amarok	Wolf	M	04/04/2012	1.6	4.7
Kenai	Wolf	M	01/04/2010	3.6	6.6
Geronimo	Wolf	M	02/05/2009	4.5	7.3
Yukon	Wolf	F	02/05/2009	4.6	7.3
Wamblee	Wolf	M	18/04/2012	Not Tested	4.5
Nanuk	Wolf	M	28/04/2009	4.5	7.3
Una	Wolf	F	07/04/2012	1.6	4.3
Chitto	Wolf	M	04/04/2012	1.6	4.3
Tala	Wolf	F	04/04/2012	1.7	4.3
Kaspar	Wolf	M	04/05/2008	5.6	8.6
Kay	Wolf	F	22/04/2012	1.5	Not Tested
Aragorn	Wolf	M	04/05/2008	5.6	8.3
Shima	Wolf	F	04/05/2008	5.6	8.4
Nia	Dog	F	22/07/2011	Not Tested	5.0
Kilio	Dog	M	18/12/2009	3.8	Not Tested
Gombo	Dog	M	21/03/2014	Not Tested	2.4
Sahibu	Dog	M	21/03/2014	Not Tested	2.4
Maisha	Dog	M	18/12/2009	3.9	6.6
Rafiki	Dog	M	30/11/2009	4.0	Not Tested
Binti	Dog	F	15/09/2010	2.2	5.9
Asali	Dog	M	15/09/2010	3.1	5.9
Bora	Dog	F	02/08/2011	2.3	Not Tested
Banzai	Dog	M	02/04/2014	Not Tested	2.4
Meru	Dog	M	01/10/2010	3.0	5.8
Hiari	Dog	M	21/03/2014	Not Tested	2.4
Imara	Dog	F	21/03/2014	Not Tested	2.4
Nuru	Dog	M	24/06/2011	2.4	4.9
Zuri	Dog	F	24/06/2011	2.4	5.1
Layla	Dog	F	03/08/2011	2.3	5.1
Pepeo	Dog	M	02/04/2014	Not Tested	2.3
Panya	Dog	F	02/04/2014	Not Tested	2.4
Enzi	Dog	M	02/04/2014	Not Tested	2.3

All wolves were born in captivity in North America and Europe. Dogs born before 2014 were obtained from animal shelters in Hungary (Tierheim Szeged and Tierheim Paks). The remaining dogs (2014 generation) were offspring of two of our own females (Layla and Nia) and were born at the Wolf Science Center. All animals except the 2014 dog cohort were separated from their mothers within 10 days of birth and then hand-raised with conspecifics in peer groups (dogs and wolves were raised separately and at different times). In the first 5 months of their life, the animals had continuous access to humans, who bottle-fed and later hand-fed them. The 2014 dog cohort spent most of the day with the hand-raisers and in peer groups but returned to their mothers at night. All animals were kept indoors during the first weeks of puppyhood and had free access to a 1,000 m^2^ outdoor, “puppy enclosure” from their second month on. They were moved to 2,000–8,000 m^2^ “living enclosures” at five months of age.

All enclosures are equipped with trees, bushes, logs, shelters and permanent sources of drinking water All animals voluntarily participate in cognitive and behavioural experiments, and/or training, and/or other social events at least once a day, and hence have daily social contact with humans. Animals are rewarded with food for participating in these activities (see section “Subjects’ Diet and Food Types”). This routine ensures that all animals are cooperative and attentive towards humans and allows regular veterinary checks without sedating the animals. All animals at the WSC are intact and males are vasectomised.

The two-choice task was conducted from October to December 2013. Of the available test subjects at that time, one wolf (Wamblee) and one dog (Nia), could not be tested because they dropped out in the training stage. The cafeteria paradigm was conducted from August to December 2016. Of the available animals at that time, one dog (Bora) had to be excluded from testing as she would not approach the test apparatus without a trainer being close (and potentially influencing the choice). Two dogs (Kilio and Rafiki- rehomed) and one wolf (Kay-deceased), that had participated in the two-choice task, could not participate in the cafeteria paradigm as they were no longer at the WSC. Dogs born in 2014 participated only in the cafeteria paradigm.

### Subjects’ Diet and Food Types

Wolves and dogs at the WSC receive a variety of foods ranging from raw meat to dog kibble both, as a part of their meals and as rewards for participating in behavioural tests. Five different food types, all equally familiar to animals, were chosen for the tests. Four foods were used in both the two-choice task and the cafeteria paradigm: 1) dead, one-day old chicks (cut into two or three pieces), 2) fresh, cow head-meat, 3) commercially available sausage (Aro Extrawurst), 4) commercially available dry food (Royal Canin–German Shepherd); and one food was used only in the cafeteria paradigm (commercially available unflavoured Tofu).

These foods were chosen because they are routinely used in behavioural tests, and one aim of our study was to investigate if wolves and dogs show different preferences for these food types, thereby affecting their behaviour in other experiments. We analysed the nutritional content of each food type (Table 2).

**Table 2:** Nutritional information for foods (reported on an ‘as is’ basis) used for testing preferences and as regular feed (values per 100 g of homogenized food).

	Energy (Kcal)	Dry Mass (g)	Moisture (g)	Crude Ash (g)	Crude Protein (g)	Crude Protein ÷ Dry Mass	Crude Fat (g)	Crude Fibre (g)
**Foods used in routine experiments**								
Chicks, one day old	103	22	78	2	15	0.7	4	0
Cow head-meat	290	45	55	1	17	0.4	26	0
Extrawurst	392	44	56	3	11	0.3	30	0
Royal Canin (GS)	384	92	8	8	24	0.3	19	3.8
Tofu	84	15	84.6	1	9	0.6	5	0
**Foods used as regular feed**								
Royal Canin (MA)	386	92	8	6	25	0.3	14	1.2
Rabbit	158	30	69.6	1	21	0.7	8	0
Chicken	235	43	57.3	5	16	0.4	20	0.4
Deer carcass	125	27	73	1	22	0.8	4	0

All food was cut into 2–3 cm^3^ pieces and stored separately. Dry food, bits of sausage and meat are used as rewards when the animals participate in both, behavioural experiments as well as touristic events. Bits of sausage are the most common rewards during training procedures involving shaping. Dry food (Royal Canin–German Shepherd) is used as the most common reward during touristic events while bits of sausage, meat and chicks are rarer treats. Once a week, animals have an enrichment session where each pack is shifted out of their enclosures and a mixture of various foods is scattered and hidden in their home enclosures for them to search for and consume. The regular feeding regimes of our animals are based on their natural feeding patterns. Dogs receive dry food (Royal Canin–Medium Adult) as an evening meal at the end of every day while wolves (as well as dogs, albeit less frequently) receive dead chickens, rabbits or pieces of deer, calf or sheep carcasses twice or thrice a week, depending on body condition, season, etc. The somewhat different feeding regimes and food quantities delivered (smaller more frequent feeding of the dogs compared to wolves) are based on their natural requirements and aimed at insuring the animal’s health.

#### Food sources

One day old chicks were obtained from “naturaldogs der Naturfuttershop”, Einsiedlingerstraße 26, 4655 Vorchdorf (47°59'05.1" N 13°55'55.0" E). The cow head-meat was purchased from “Fleischerei Pfennigbauer Hausleithen”, Hauptplatz 17, 3464 Hausleiten (48°23'45.1" N 16°06'06.7" E). The Sausage (Brand: Aro, 1.5 kg packs) was purchased from METRO Cash & Carry Austria GmbH, Wiener Straße 176–196, 2103 Langenzersdorf (48°17'52.4" N, 16°22'22.7" E). Royal Canin Österreich GmbH, Handelskai 92, Rivergate/Gate 1/OG 11, 1200 Wien (48°14'32.5" N 16°23'04.9" E) supplied all dry food (Type: Medium-Adult and Adult “German Shepherd”, 12 kg packs). Tofu (Brand: Zurück zum Ursprung, 150 gm packs) was purchased from Hofer Kommanditgesellschaft, Wienerstraße 1, 2115 Ernstbrunn (48°31'34.9" N, 16°22'44.6" E). Rabbits were supplied by Baxter, Uferstrasse 15, 2304 Orth/Donau (48°08'07.5" N 16°42'28.4" E). The Ernstbrunn Wildpark provided the deer carcasses, some chickens and some rabbits. All animals used as feed were obtained dead and were not euthanised at the Wolf Science Center.

### General procedure

For each task, a training phase preceded the testing phase. As the experimental setups were novel to the subjects, they were trained to operate each apparatus. Subjects were trained with positive-reinforcement (with the aid of a clicker). Bits of sausage and dry food were used as rewards during the shaping process. Subjects were tested once they had reached objective, task-specific criteria for being considered “trained” (see below) in each task. The number of training sessions required for a subject to reach criteria relied solely on the subject’s performance.

Subjects were tested under two conditions: “high satiation” (henceforth called “fed”) and “low satiation” (henceforth called “unfed”). For the fed condition subjects were fed approximately 15 hours prior (i.e. the previous evening) to the test session. Wolves were fed either one complete rabbit each or similarly sized portions of a deer carcass. Dogs were fed their regular measures of dry food (different from the food used for the test). For the unfed condition, the wolves were not fed the evening before testing. Two dogs could not be kept completely unfed overnight due to medical reasons. To ensure consistency, all dogs were fed less than half their regular measures of dry food approximately 15 hours prior (i.e. the previous evening) to the test session.

The testing phase for the two-choice task consisted of four sessions each in the fed and unfed conditions. Each session consisted of twelve trials (two trials for each of the six possible combinations of the four foods that were used). The testing phase for the cafeteria paradigm consisted of two sessions each in the fed and unfed conditions. Each session consisted of five trials. In both tasks, we performed only one session per subject per day.

## Two-choice task

### Apparatus

**Fig 1:**
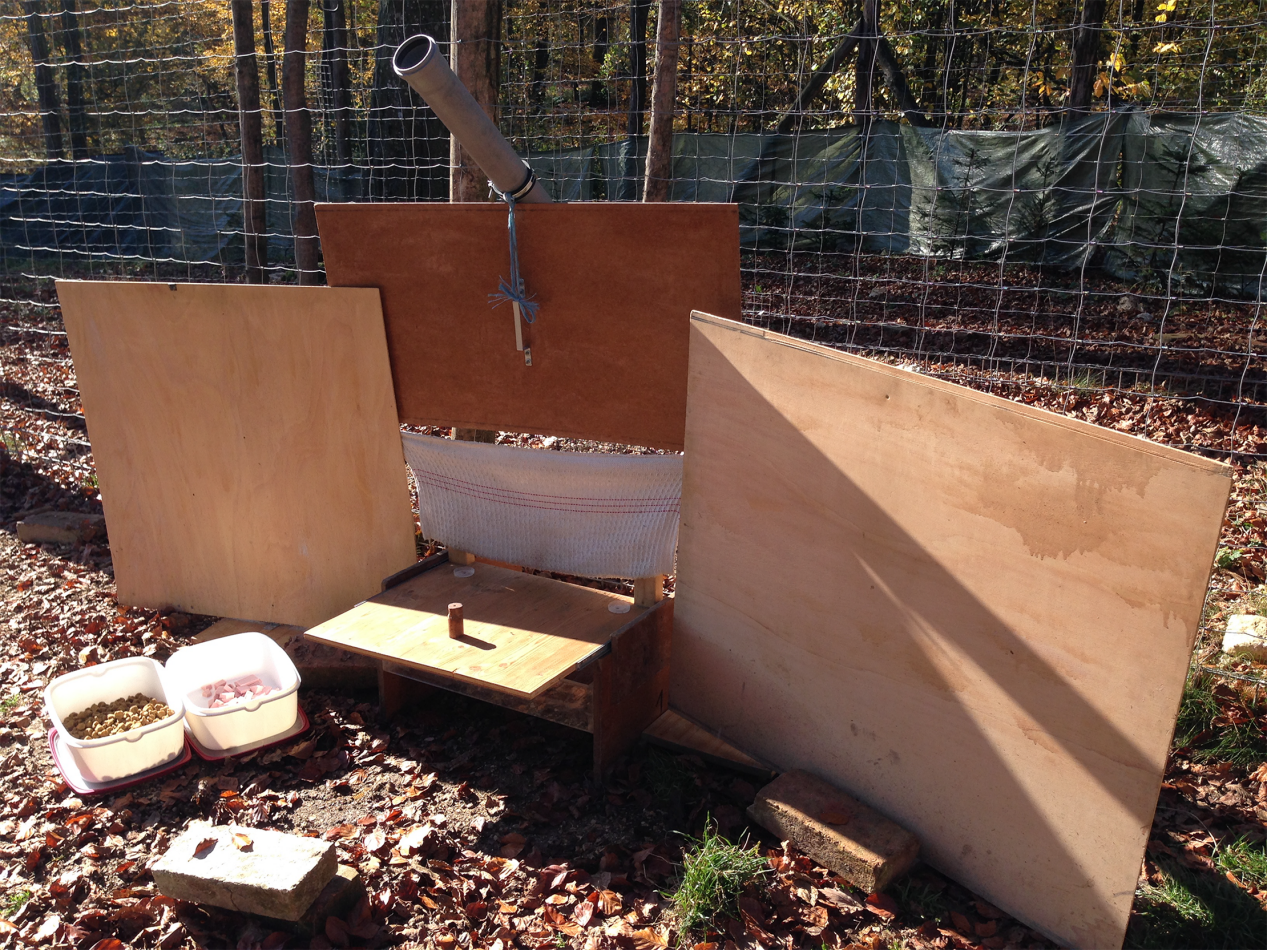
Apparatus used for testing food preferences in the two-choice task (rear).

The apparatus consisted of a low table (57.5 cm × 49.3 cm) with the following features: a sliding “choice tray” with two wooden blocks (henceforth called “targets”, sized 14.7 cm × 5.5 cm × 3.3 cm) fixed to its left and rightmost extremes (on the side that would be closest to the animal) mounted on top of the table, and a flexible plastic tube (henceforth called the “chute”) attached to the central part of the table. The experimenter could deliver food to the subject via the chute. A panel with flaps hid the experimenter from view of the subject while allowing food to be passed through (See Fig 1). A single, central food delivery system was chosen to reduce the chances of the subject developing a side bias.

### Experimental setup

The experiment took place in an outdoor testing enclosure. The subject was positioned inside a shifting channel in the test enclosure and was free to move around. The apparatus was positioned outside the shifting channel. The experimenter was positioned outside the shifting channel, behind the apparatus and was occluded from the subject’s view by the apparatus’s flaps. A trainer was positioned behind the experimenter and was visible to the test subject. Two subjects (Una and Kay) were not comfortable with the experimenter, therefore a second trainer adopted the role of the experimenter for these subjects.

### Procedure

#### Training phase

Training was divided into three sub steps for the two-choice task. The criterion for a subject to proceed to the next training step was scoring nine out of twelve trials correctly in two sessions. Subjects were first trained to touch a target with their nose. Next, training sessions consisting of four “warm-up” trials and one to two sets of twelve single choice trials were performed. The number of single-choice trial sets depended on the motivation of the subject during that training session. Subjects had only one training session per day. During a warm-up trial, a trainer presented food to the subject twice on each side. In a single choice trial, an experimenter showed the subject a piece of food in the middle of the table and placed it in a small cup on one of the sides of the sliding platform, leaving the other cup empty. The order in which the food was presented on the left or right was semi-randomised such that the food was not presented on the same side more than twice in a row. The sliding platform was then extended, allowing the subject to nose one of the targets. Nosing the target adjacent to the food item was considered a “correct” choice. If the subject chose correctly, the experimenter retracted the platform, picked up the food, showed it to the subject and delivered it to the subject via the chute. In case of an incorrect choice, the experimenter retracted the platform and repeated the trial with the food on the same side.

The second step in training involved removing human cues from the setup. The experimenter now baited both cups on the platform with identical pieces of food, out of view of the subject and extended it partially. The subject was given three seconds to inspect the food after which the platform was extended fully. The subject could then touch either target with its nose to obtain the food on the corresponding side.

The aim of the third step was to allow the subject to understand that the food that was not chosen first was no longer available. The procedure was identical to that of step two, except that each side was baited with a different food item. The location of each food type was semi-randomised such that it was not presented on the same side more than twice in a row. If a subject displayed a side bias (i.e. chose food on the same side in all 12 trials), a “correction” session was performed, in which the subject was given a choice between dry food and no food in the same semi-randomised fashion. The criterion for a subject to proceed to the testing phase was that it did not show a side bias in the third training step.

#### Testing phase

Each test session began with two single-choice trials to ensure the subject was still familiar with the working of the apparatus. The test procedure was identical to step three of the training phase. A test session consisted of twelve trials, two for each of the six possible combinations of food. The order of presentation of the dyads was semi-randomised so that the same pair of food choices did not occur more than twice in a row. Each subject had only one test session per day. Each subject had four test sessions before feeding and four sessions after feeding. See S1 Video for an example of a trial.

### Analyses

Data for the two-choice task were analysed using generalised linear mixed models with Poisson distributions fit by the Laplace approximation. We used the package “lme4” [48] in R (v 2.14.1) [49]. We tested the effects of species, satiation state, sex and food type on the frequency of choice of food. To evaluate whether wolves’ and dogs’ preference varied depending on food type and whether satiation levels affected food choice differently in wolves and dogs, we included a species by food type, and a species by satiation state interaction in the model. “Individual” was added as a random effect and analyses were normalised for the number of presentations. To better understand the effects that we found in the overall analyses, we used generalised linear mixed models with the binomial distribution to test the effects of species, satiation state and sex on the likelihood of choosing a food for each of the six combinations the subjects were presented with (i.e. chicks and meat, chicks and sausage, chicks and dry food, meat and sausage, meat and dry food, and sausage and dry food). We adopted a model reduction approach based on p-values and starting with interactions. One individual (Nanuk) was excluded from the analyses as he did not consume the food after choosing it.

## Results

**Fig 2:**
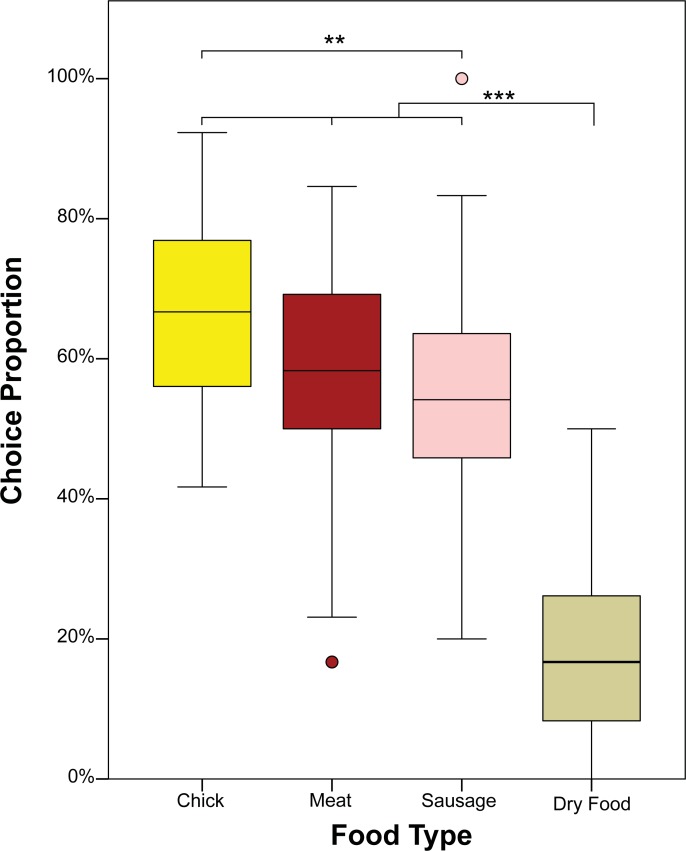
Percentage of food choices, normalised by number of presentations. Circles indicate outliers. Two asterisks indicate a statistically significant difference with P < 0.05 and three asterisks indicate a statistically significant difference with P < 0.001.

Dogs and wolves did not differ in the frequency with which they chose specific foods (species by food type interaction: F = 1.72, P = 0.2) and did not choose differently whether fed or unfed (species by satiation state: F = 0.14, P = 0.7). Furthermore, there were no main effects of sex (F = 0.34, P = 0.6), satiation state (F = 0.06, P = 0.8) or species (F = 0.39, P = 0.5). The frequency of choice was influenced by food type (F = 92.3, P < 0.001): sausage was chosen less often than chick (F = 2.302, P = 0.021), but was not chosen significantly differently from meat (F = 1.518, P = 0.129). No difference emerged in the frequency of choosing chicks and meat (F = 0.798, P = 0.425), but dry food was chosen least often compared to all other food types (dry food: vs. chick F = 11.043, P < 0.001; vs. meat F = 10.477, P < 0.001; vs. sausage F = 9.297, P < 0.001) (Fig 2). To see species-wise distributions of choice proportions and for pairwise comparisons between each food type, see S1 File.

## Cafeteria paradigm

### Apparatus

The apparatus consisted of six transparent, perforated Plexiglas boxes measuring 20 cm on each side, mounted on an arch-shaped wooden platform (55 cm wide, 5 cm tall and approx. 120 cm in diameter) (Figure A in S2 File). Commercially available stainless-steel dog-food plates measuring 8 cm in diameter were placed under each Plexiglas box and were fastened to the platform using a screw. Each plate was 75 cm away from the ones adjacent to it. The Plexiglas boxes were mounted with hinges on one side in a way that they could be flipped open. All boxes could be remotely locked, making them impossible to open. Each food plate was used only for a single type of food to prevent potential mixing of food odours and flavours. During the test, a visually equal amount of each food (one to two pieces of meat, sausage, chicks and tofu and four to five pieces of dry food) was used for baiting the boxes.

### Experimental setup

**Fig 3:**
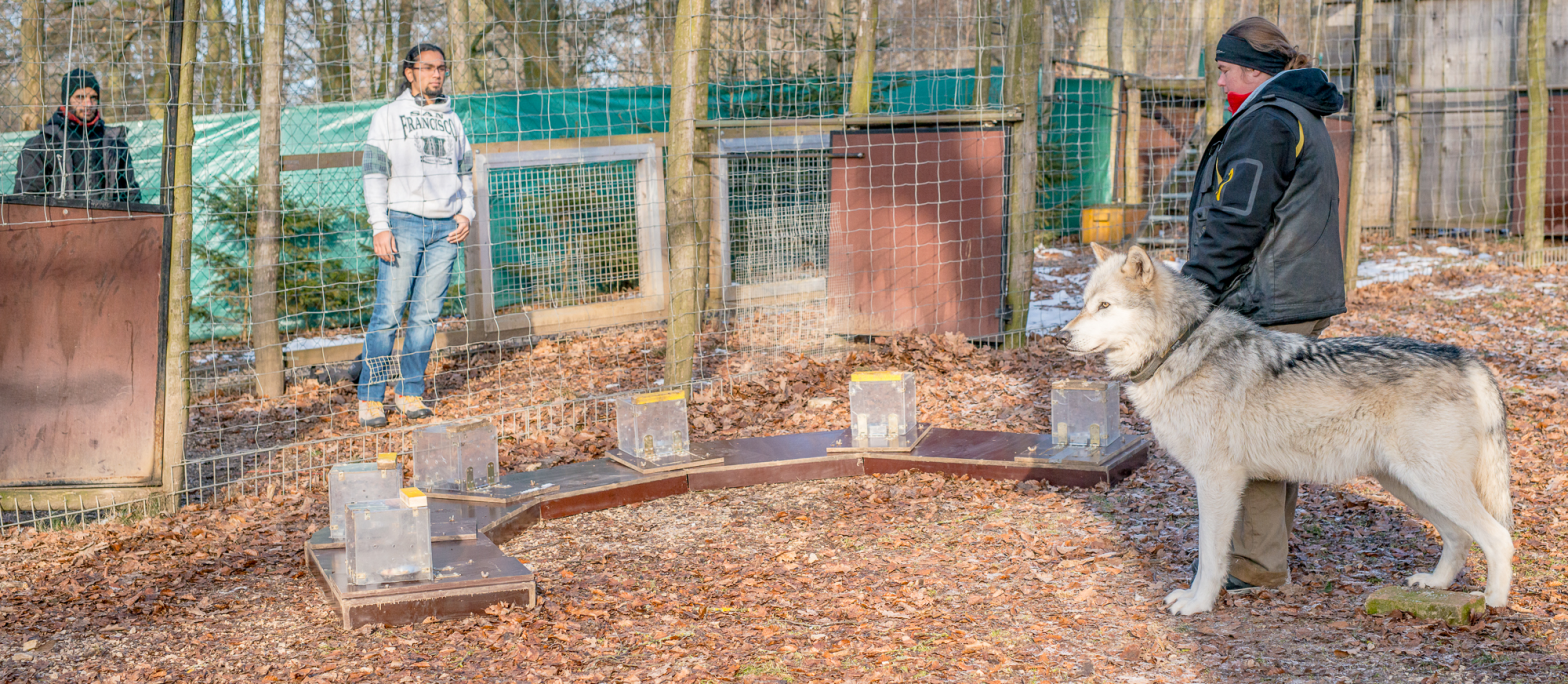
Experimental setup for the cafeteria paradigm. (Left to right) helper, experimenter, trainer and test subject.

The experiment took place in an outdoor testing enclosure. A trainer stood with the subject on a marked spot in the concave part of the arch such that each box was equidistant from the test subject. The experimenter was positioned outside the testing enclosure but inside a shifting channel, in sight of the subject, and re-baited the apparatus between trials (see Fig 3). A helper operated the shifting mechanism and let the experimenter in and out of the testing enclosure. One subject (Una) was not comfortable with the experimenter and helper therefore two additional trainers adopted these roles for this subject.

### Procedure

#### Training phase

Each subject received at least one habituation and/or training session to familiarize it with the mechanism of the apparatus and to associate the apparatus with food. A small part of the wooden platform (measuring approx. 100 × 55 cm) containing just one Plexiglas box was used for these sessions. The entire setup was not used to prevent the subjects from developing any preferences for a specific position.

Subjects were trained to flip the Plexiglas box open using their paw or snout by shaping with a clicker. All five food types were used to bait the apparatus during training to prevent the subjects from associating the mechanism with a specific kind of food. Bits of dry food and sausage were used as rewards during the shaping process. The objective of the experiment was not to test problem solving abilities but to assess food preferences. Hence, in cases where the subjects were overly fearful of the movement of the Plexiglas box or in cases where the subjects could not learn to open the boxes on their own after three sessions, subjects were trained to indicate their choice by placing their paw on the apparatus, following which, the trainer flipped open the box for them (10 dogs and 8 wolves indicated at least once in four sessions; 5 dogs and 3 wolves indicated in all sessions). Subjects were considered trained once they required no cues from the trainer and flipped the Plexiglas box open themselves (or placed their paw on the box signalling the trainer to open the box) at least four out of five times the box was baited.

#### Testing phase

To prevent potential confounding effects of previously eaten foods, the subjects participated in this experiment prior to participating in any other tests that day. For the test, the subject was either walked to, or shifted (via a series of shifting channels) into the test enclosure, where the un-baited apparatus was present. Subjects were given 2 to 5 minutes to explore the enclosure and inspect the apparatus. This was done to minimise the number of distractions during the test session.

A test session consisted of one inspection phase and five test trials. Two test sessions were conducted in each satiation condition. The position of foods was changed between sessions but remained constant across trials within each session. Every session was recorded with a video camera mounted on a tripod positioned behind the first fence beside the experimenter. Data for choice of food and duration of attempts to make a choice or extract food when the apparatus was locked were extracted from the recorded videos.

The trainer then called the subject back and held it on a leash or by a collar while the experimenter entered the test enclosure and baited each box with different food item. One box was left empty and served as a control. The order in which the boxes were baited was randomised. Once baited, the boxes were locked remotely, and the experimenter exited the enclosure.

**Inspection:** The trainer then walked the animal to each box and allowed the subject to see and sniff each food box. In case the subject was distracted, the trainer called out to the subject, pointed to each box and ensured that the animal had seen and sniffed it (Figure B in S2 File). At this point, the animal could not open the boxes and access the food. The order in which the trainers had subjects inspect the boxes and the box the subjects inspected first was randomised. The trainer then walked the subject back to a marked position from which all foods were equidistant from the subject.

**Test trial:** A trial started with the boxes being remotely unlocked and the subject being released by the trainer from the marked position.

All subjects could open a maximum of three boxes and could eat the food under each, after which, the remaining boxes were locked remotely, and the subject was called back by the trainer, ending the trial. At the end of a trial, subjects were rewarded with bits of dry food for returning to the trainer. See S1 Video for an example of test trials.

**Rebaiting:** After each trial, the experimenter entered the test enclosure and replaced the food which the subject had consumed. The experimenter pretended to rebait the boxes that still had food under them to prevent potential local enhancement effects. The order in which boxes were rebaited / mock-rebaited was randomised.

### Behavioural coding

Videos were coded using Solomon Coder beta v. 17.02.15 (a behaviour coding software developed by András Péter, Dept. of Ethology, Budapest). See Table 3 for definitions of behaviours coded and S1 Video for examples of coded behaviours.

**Table 3:** Definitions of coded behavioural elements.

Action	Definition
Release	The subject starts moving towards the apparatus after the trainer releases it; the subject is now free to approach the apparatus and make a choice.
Choice #	The subject either flips a box open with its snout or paw, attempts to flip it open more than once or places its paw on or in front of a box indicating that the trainer should open it, followed by the trainer opening the box.
Extra Attempt	The subject attempts to open a box by indicating, pawing, biting, scratching or pulling at it either during”Inspection” or after Choice 3.

### Analyses

Food choice data for the cafeteria paradigm were analysed using a Generalised Estimating Equation (GEE) with a multinomial distribution and cumulative logit link in SPSS (v 23.0). For each choice, we tested whether food choice could be predicted by species, satiation state or an interaction between the two. To better understand how each food type contributed to the effects found in the overall model, we further analysed each food type separately. We tested whether the likelihood of choosing each food type could be significantly predicted by species, satiation state or an interaction between the two (GEE, binomial distribution with a logit link). When analysing Choice 2, we also tested for the effect of foods being adjacent to the previous choice. We accounted for the change in the food types available by factoring Choice 1 into the model. This also allowed us to analyse whether any of the food types chosen first affected the second choice. We were unable to analyse Choice 3 as we did not have enough data to compute the model reliably after controlling for both Choice 1 and Choice 2. We have hence reported only the results for the first two choices.

We calculated each subject’s choice diversity in each satiation state (by pooling their choice data in each trial in both sessions) using Shannon’s diversity index [50]. Diversity index data were analysed using linear mixed effects models fit by maximum likelihood with the package “lme4” (v 1.1–13) [48] in R (v 3.4.1) [49]. We tested whether choice diversity could be significantly predicted by choice order (whether it was the first or second choice), species or satiation state. We tested interactions between species and satiation state, species and choice order and satiation state and choice order.

We calculated the duration subjects attempted to make “additional” choices in either the initial, inspection phase or after making the three permitted choices (“Extra attempts”- Table 3). These data were analysed using generalised additive models for location, scale and shape with the package “gamlss” (v 5.0–5) [51] in R (v 3.4.1). Data distributions were identified using the “gamlss.Dist” package (v.5.0–3). We used a GAMLSS model with the generalised inverse Gaussian distribution to test whether the duration of extra attempts could be significantly predicted by species, satiation state or an interaction between the two. We adopted a model reduction approach selecting models by minimising their generalised Akaike information criteria [52].

## Results

**Fig 4:**
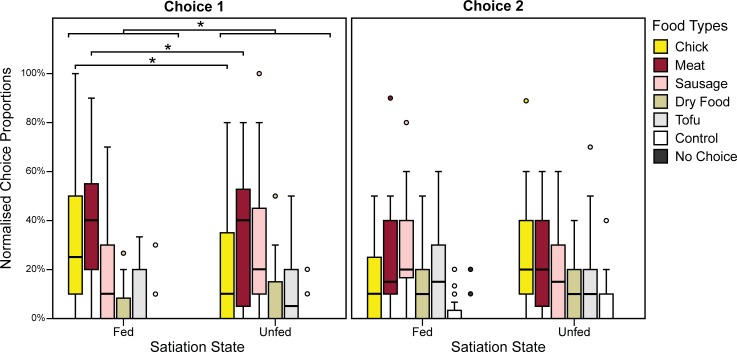
**Proportion of food-types chosen first (left) and second (right) in each satiation state, normalised by number of trials**. Circles indicate outliers and asterisks indicate statistical significance at α = 0.05.

We found an effect of satiation state (Wald χ^2^ = 4.7, P = 0.03) but not species (Wald χ^2^ = 1.09, P = 0.296) on the proportion of food-types chosen (Fig 4). The interaction between species and satiation state was not significant (Wald χ^2^ = 0.721, P = 0.396).

A closer analysis of each food type individually (see Table 4 for a summary of results) showed that the effect of satiation state was driven mainly by two factors: (1) Subjects (both wolves and dogs) chose chicks significantly less when unfed than fed (Wald χ^2^ = 4.449, P = 0.035) (Fed: 30%, Unfed: 18.18%); (2) Dogs and wolves chose meat differently between satiation states (Wald χ^2^ = 5.33, P = 0.021). A Post hoc Estimated Marginal Means analyses (Wald χ^2^ = 11.77, P = 0.008) revealed that wolves chose meat 20% less when unfed (Holm-Bonferroni corrected P = 0.005) than when fed, while dogs did not differ in the proportion of meat chosen between satiation states.

**Table 4:** Factors predicting the likelihood of a food being chosen as the first choice.

Food Type	Species		Satiation state		Species*Satiation state	
	Wald χ^2^	P	Wald χ^2^	P	Wald χ^2^	P
Chick	0.768	0.381	4.627	0.031*	1.683	0.194
Meat	0.002	0.969	1.025	0.311	5.126	0.024*
Sausage	0.241	0.623	2.961	0.085	0.73	0.787
Dry Food	0.001	0.979	2.573	0.109	1.905	0.168
Tofu	1.114	0.291	0.949	0.330	0.191	0.662

We found no effect of either species (Wald χ^2^ = 0.231, P = 0.631) or satiation state (Wald χ^2^ = 3.094, P = 0.079) on the proportion of food chosen by subjects as their second choice. The interaction between species and satiation state was not significant (Wald χ^2^ = 1.926, P = 0.165). Overall, foods chosen in the second choice were not predicted by their proximity to the first choice (Wald χ^2^ = 2.254, P = 0.133). There were no significant interactions between the proximity to the first choice and species (Wald χ^2^ = 2.001, P = 0.157) or first choice and satiation state (Wald χ^2^ = 0.006, P = 0.936). The second choice was significantly affected by the first choice but only if Sausage (and not other food types) was chosen as first choice (Wald χ^2^ = 5.486, P = 0.019). For results of analyses for each food type, see S2 File, for complete model information and parameter estimates for the first choice, see S3 File and for complete model information and parameter estimates for the second choice, see S4 File.

**Fig 5:**
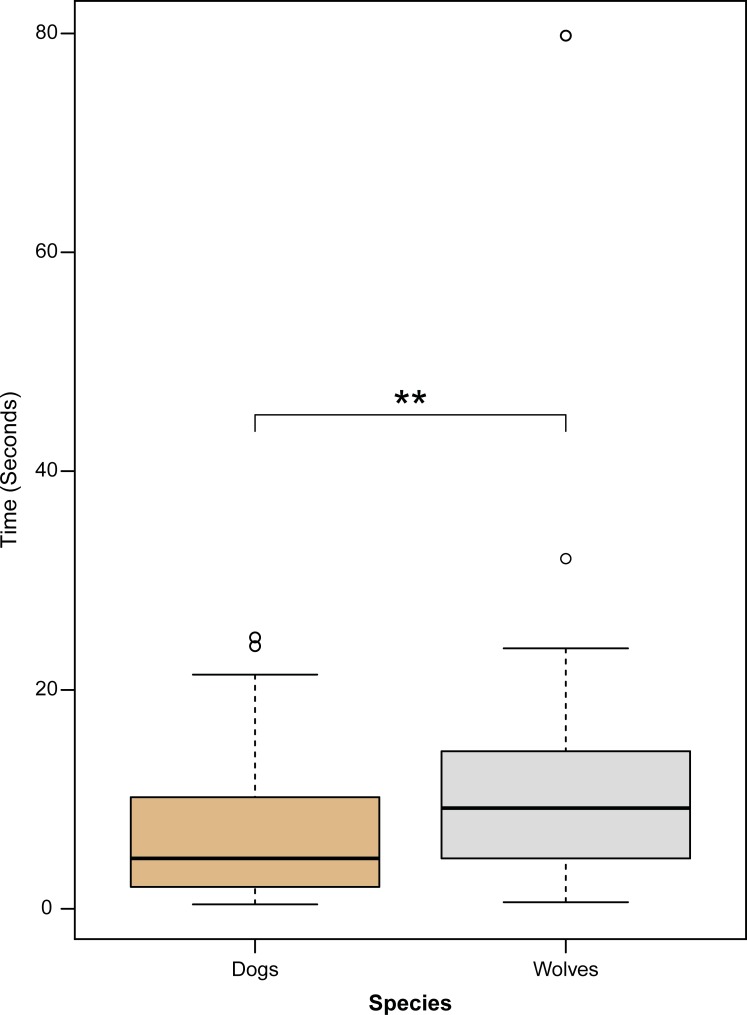
Duration of time subjects spent attempting to obtain inaccessible food. Circles indicate outliers and asterisks indicate statistical significance at α = 0.05.

The interaction between species and satiation state did not have a significant effect on the time subjects spent attempting to get food outside of the permitted choices (t = 0.238, P = 0.514). Overall, regardless of satiation state (t = 0.897, P = 0.372), wolves spent more time than dogs (t = 2.874, P = 0.005) attempting to obtain extra food (median duration wolves = 9.2 sec, dogs = 4.6 sec) (Fig 5).

There was a significant interaction between species and satiation state on the choice diversity (t = 2.511, P = 0.013). To better understand this interaction, we analysed the effect of satiation state on choice diversity separately for each species. In dogs, choice diversity did not vary significantly between satiation states (t = -0.984, P = 0.348), but in wolves, choice diversity was significantly higher in the unfed condition (t = 2.286, P = 0.028). When unfed, dogs and wolves did not significantly differ in their choice diversity (t = 0.081, P = 0.936) but when fed, wolves were significantly less diverse in their choices than dogs (t = -2.66, P = 0.013).

**Fig 6:**
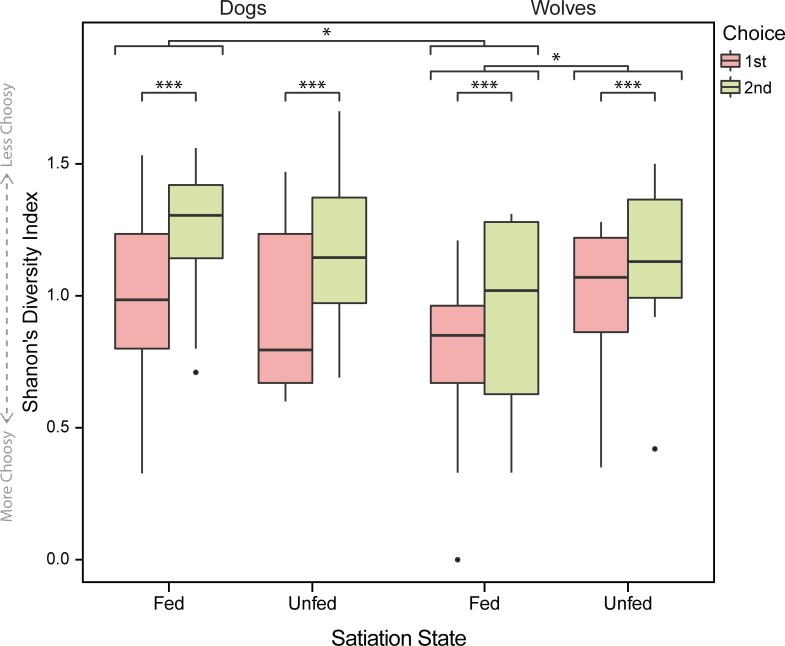
Choice diversity indices across choices, split by species and satiation state. Circles indicate outliers and asterisks indicate statistical significance in comparisons at α = 0.05 (*: P < 0.01, ***: P < 0.001).

Overall, choice diversity was significantly lower in the first choice than in the second choice (t = 3.60, P < 0.001) (Fig 6). The interactions choice order by species (t = -0.691, P = 0.491) and choice order by satiation state (t = 0.176, P = 0.861) were not significant.

## Discussion

The current study aimed to investigate the food preferences of dogs and wolves, and to this end, we conducted food preference tests in two satiation states with two different paradigms.

Overall, we found only minor differences in dogs’ and wolves’ food preferences. Contrary to our prediction that dogs would show a weaker preference for meat over kibble rich in starch, dogs and wolves did not differ in their preference for meat in either testing paradigm. In the cafeteria paradigm, both dogs and wolves chose nearby foods in the same manner. The only observed difference related to choice diversity, where wolves were less diverse (or more “selective”) than dogs in the fed condition. Overall it appears that dogs’ and wolves’ different foraging styles have not affected their food preferences. While dogs’ better starch digestion has been proposed to be an early effect of their domestication [22], recent studies suggest that this adaptation may have occurred later than previously thought [23]. Of course, “absence of evidence is not evidence of absence”, and it is still possible that dogs’ and wolves’ foraging styles have affected their food preferences, but that these differences are overshadowed by stronger factors, such as shared feeding habits and food availability of our captive animals, or that the relatively small sample size does not allow for such differences to emerge. Human food preference tests have shown that preference patterns can be affected by previously consumed meals [53]. Prior to the “fed” condition, dogs were fed kibble and wolves were fed carcasses (see Methods). It is possible that this may have caused dogs’ preference for chicks and meat to increase (and wolves’ preference to decrease) the following day and buffered potential differences in dogs’ and wolves’ food preferences. However, this is unlikely considering we found no differences in dogs’ and wolves’ preferences in either feeding condition.

We found similar patterns in wolves’ and dogs’ food preferences in both paradigms: three kinds of food were chosen the most by both dogs and wolves, namely chicks, meat and sausage. Nutritive value may be one of the explanatory factors for this pattern. The high choice proportion of protein-rich chicks (after correcting for dry mass) is partly in line with the work on macronutrient selection [3–6]. However, tofu had (corrected) protein content comparable to chicks and higher than meat and sausage, and the kibble had a higher calorific value than all three of the other foods. Yet, tofu and dry food were chosen the least often. If nutritive value was the sole explanatory factor, protein-rich and high-energy foods should have had comparable choice proportions. That they did not could indicate that the hedonic quality of food (taste/flavour) may override nutritional value. Perhaps subjects avoided foods with extremely low or extremely high moisture contents, which is why tofu and kibble had low choice proportions. Alternatively, the high choice proportions of meat and sausage may have been influenced by their fat content (the highest from the foods we used). Dogs and wolves (like humans and several other animals [54]) may have evolved a preference for fatty foods which may have influenced this choice pattern.

One of the most important factors that emerge in determining the animals’ choice is the rarity of the most chosen foods in the subjects’ daily diet (chicks being the rarest, followed by meat and then sausage). Here, it is also interesting to note that because of the different health requirement of wolves and dogs, their daily feeding regime is somewhat different: dogs receive a higher proportion of dry kibble (which is their staple diet) and only little meat from carcasses, whereas it is the opposite in wolves. ‘Rarity’ could have been a stronger motivating factor for dogs than wolves. Further, as mentioned earlier, tastes of previously consumed meals can affect subsequent food choices in humans [53] and perhaps even in animals: dogs’ and wolves’ different feeding regimes could have influenced their food preferences in our tests (for example, consuming kibble as a meal the evening prior to a test may increase preference for meat in the test and vice-versa). Yet, no substantial differences emerged in the wolves’ and dogs’ choices.

Satiation did not affect food choice in the two-choice test and only moderately did so in the cafeteria paradigm. In the latter, subjects were significantly less likely to choose chicks as the first choice when unfed. The proportion of sausage as first choice increased marginally. However, it is noteworthy that when ‘unfed’, subjects still chose meat and/or chicks as their second choice even when they were not ‘nearby’ foods. This suggests that subjects sought these foods out, supporting results showing that these were indeed their preferred foods. In contrast, dry food and tofu were more likely to be chosen when they were nearby foods. It is likely that these choices were made impulsively immediately after Choice 1. However, these effects of satiation were not evident in the two-choice task. While widely used [41,42], this task is known to have shortcomings [44]. In our case, it is likely that task contingencies such as side biases or experiences with similar, two-choice tasks that subjects participated in earlier (such as the numerical competence task [55], for example) may have overshadowed the effects of satiation in the two-choice food preferences task. By allowing multiple choices, the cafeteria paradigm allows the construction of a preference scale of foods, which can then be understood further with diversity indices. In fact, subjects were significantly more diverse when making their second choice than their first choice. This supports the idea that subjects initially sought out highly preferred foods and were not as choosy afterwards.

While choice diversity did not differ between satiation states in dogs, wolves were significantly more diverse in their choices when unfed compared to when fed. Hunger can affect foraging styles [27]. A proximate explanation for the current results, could be that wolves were more “impulsive” when hungry and paid less attention to the position of foods. Although a number of studies have been carried out comparing wolves’ and dogs’ inhibitory control, with no consistent differences emerging [56,57], satiation level is not a variable that has so far been considered in such studies. Current results suggest it may be of interest for future research; orexigenic and/or anorexigenic measures of satiety can be used to objectively quantify hunger levels [58–60].

Importantly, establishing that wolves and dogs in our facility do not differ in their preferences has significant implications for the other behavioural studies conducted at the centre. Our subjects have participated in several behavioural and cognitive experiments over their lifetime, many of which have involved food rewards [17,28,30,33,56,61,62]. For example, we found wolves to be more persistent than dogs in trying to obtain inaccessible food, a result that is in line with numerous other studies [28,30,56,63–69]. Considering the results from this experiment, we can firmly conclude that the wolf-dog differences observed were not driven by differences in wolves’ and dogs’ food preferences, but more likely due to differences in their motivational states regardless of the type of food reward.

Taken together, we found no evidence for the hypothesis that dogs’ and wolves’ foraging styles have affected their food preferences and conclude that domestication has most likely not significantly affected food preferences in dogs. Choice patterns were mildly affected by hunger in wolves but not in dogs. We suggest that the cafeteria paradigm is more sensitive in detecting such differences than a two-choice task. Finally, our results indicate that differences in our wolves’ and dogs’ performance in behavioural/cognitive tests with food rewards is not significantly affected by diverging food preferences in wolves and dogs.

## Supporting information

S1 Video Two-choice task & cafeteria paradigm procedure. (MP4)

S1 File Additional two-choice task results. (PDF)

S2 File Additional cafeteria paradigm methods and results. (PDF)

S3 File Model information for cafeteria paradigm (first choice). (DOCX)

S4 File Model information for cafeteria paradigm (second choice). (DOCX)
