# Supplementary material for: Food preferences of similarly raised and kept captive dogs and wolves
Source: PLoS One. 2018 Sep 20;13(9):e0203165. doi: 10.1371/journal.pone.0203165 (PMC6157812; doi:10.1371/journal.pone.0203165)
Supplement: S1 File — (PDF) [file pone.0203165.s002.pdf]

Supplementary Information 2: Additional Two-Choice Task Results

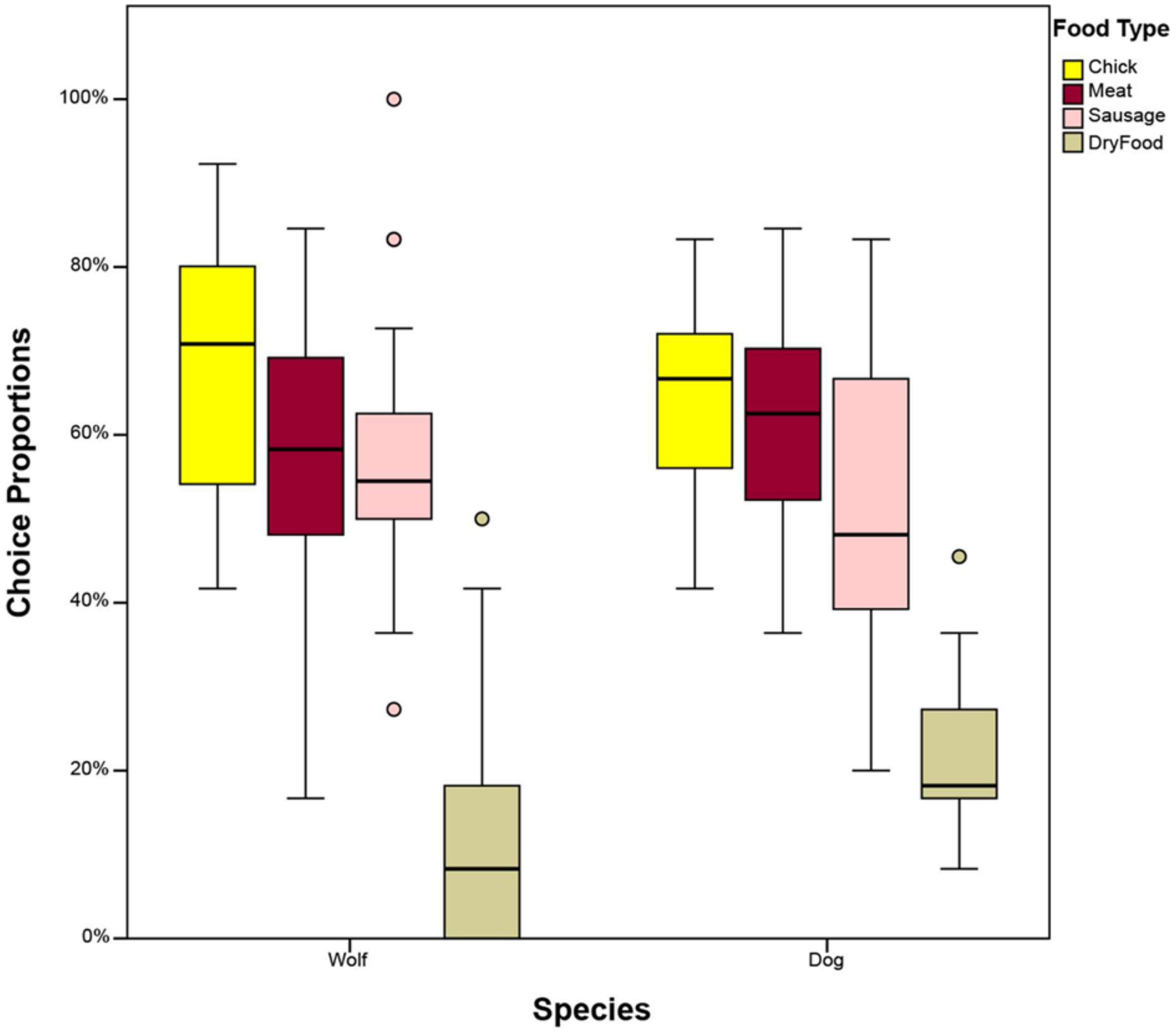

S2 Fig. Proportion of food choices, normalised by number of presentations, split by species. Circles indicate outliers

Considering each food pair separately, we found that subjects preferred chicks ($z = 7.325$, $P < 0.001$) and meat ($z = 8.461$, $P < 0.001$) to the dry food, and this preference was not affected by species (dry food: vs. chicks $F = 1.51$, $P = 0.2$; vs. meat $F = 0.62$, $P = 0.4$), sex (dry food: vs. chicks $F = 0.43$, $P = 0.5$; vs. meat $F = 0.14$, $P = 0.7$), or feeding condition (dry food: vs. chicks $F = 0.08$, $P = 0.8$; vs. meat $F = 0.39$, $P = 0.5$).

Subjects did not prefer meat or chicks significantly over each other ($z = 0.982$, $P = 0.326$) and this was not affected by species ($F = 2.13$, $P = 0.16$), sex ($F = 0.53$, $P = 0.48$) or feeding condition ($F = 2.8$, $P = 0.1$).

Subjects preferred chicks to the sausage ($z = 4.974$, $P < 0.001$) and this was not affected by species ($F = 0.03$, $P = 0.9$), sex ($F = 0.01$, $P = 0.9$) or feeding condition ($F = 6.2$, $P = 0.4$). Wolves preferred the sausage to the dry food more often than dogs ($z = 1.993$, $P = 0.046$) and both wolves and dogs preferred the sausage to the dry food more often when fed than when unfed ($z = 2.313$, $P = 0.021$). But both wolves ($z = 6.968$, $P = 0.001$) and dogs ($z = 4.816$, $P < 0.001$) preferred the sausage to the dry-food regardless of feeding condition; and preferences were not affected by sex ($F = 0.31$, $P = 0.58$).
