# Supplementary material for: Food preferences of similarly raised and kept captive dogs and wolves
Source: PLoS One. 2018 Sep 20;13(9):e0203165. doi: 10.1371/journal.pone.0203165 (PMC6157812; doi:10.1371/journal.pone.0203165)
Supplement: S2 File — (PDF) [file pone.0203165.s003.pdf]

Supplementary Information 3: Cafeteria Paradigm Methods

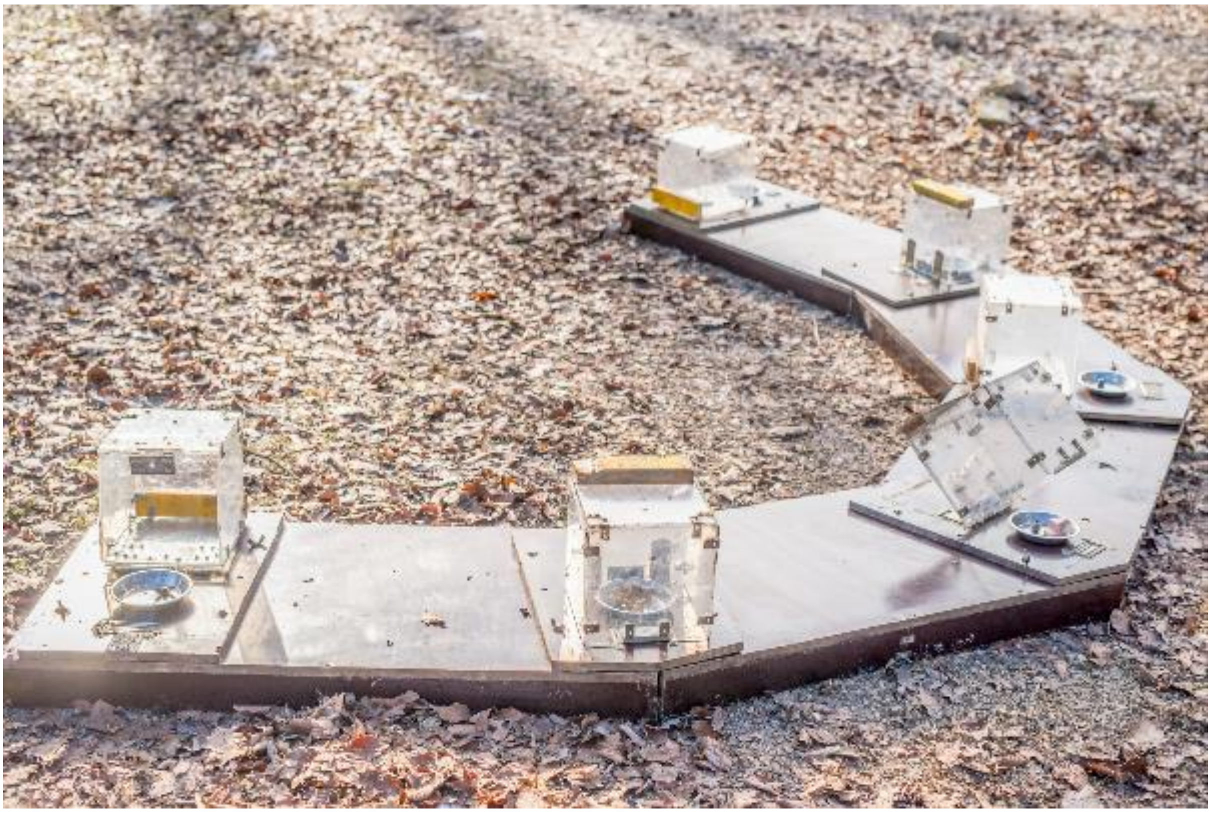

S3 Fig. A Apparatus used for the Food Preferences Test

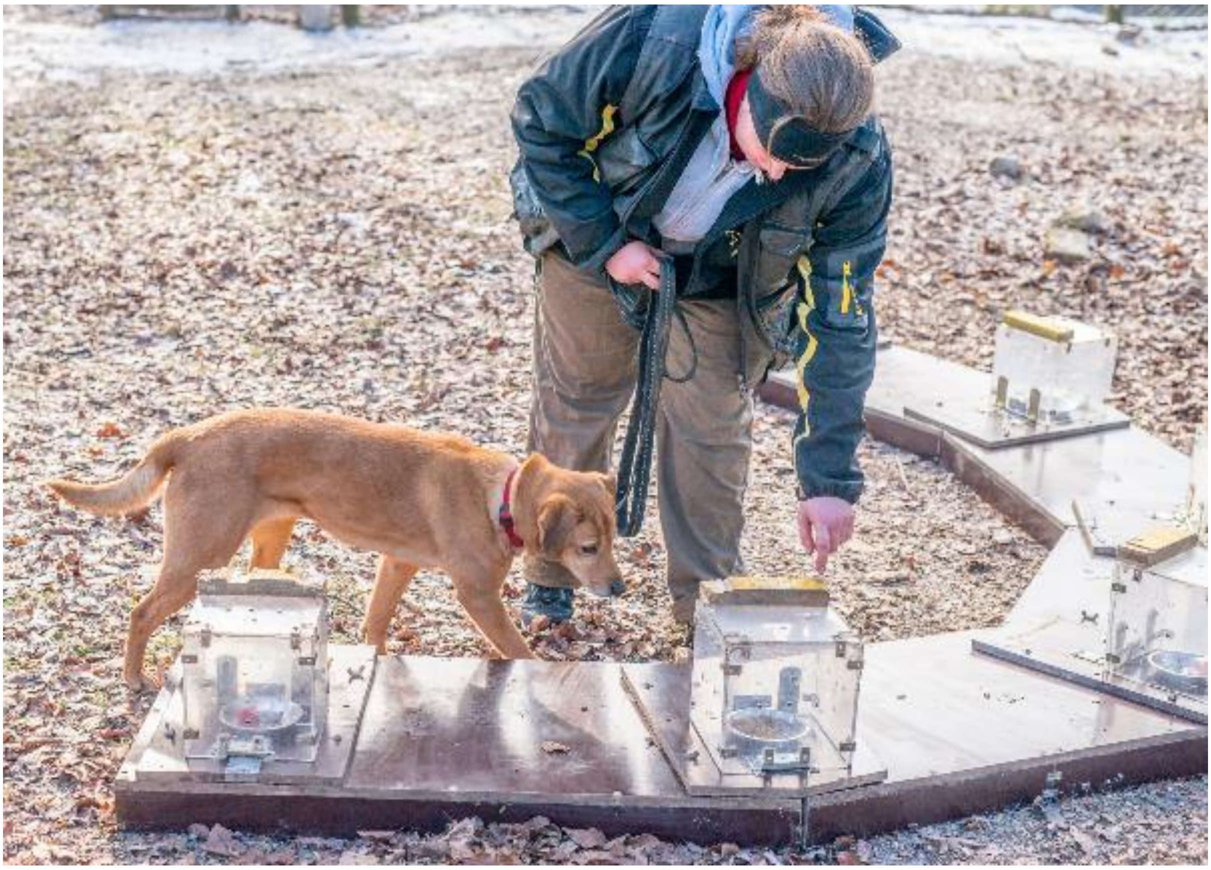

S3 Fig. B Inspection Phase: Trainer pointing at box to ensure test subject sees and sniffs it

Cafeteria Paradigm – Additional Results

A closer analysis of each food type individually (see S3 Table 1 and S3 Table 2 for a summary of results) showed that these effects did in fact, differ between food types.

A significant species effect emerged in an interaction with feeding condition in the subjects' preference for tofu. Post hoc Estimated Marginal Means analyses (Wald χ^2 = 24.174, P < 0.001) revealed that when unfed, wolves were less likely than dogs to choose tofu (Holm-Bonferroni corrected P = 0.035).

We found a significant interaction between feeding condition and proximity to the first choice in the subjects' preference for meat. Post hoc Estimated Marginal Means analyses (Wald χ^2 = 20.173, P < 0.001) revealed that in the unfed (Holm-Bonferroni corrected P = 0.008) but not in the fed condition (Holm-Bonferroni corrected P = 0.489), subjects were 33.34% less likely to choose meat when it was in proximity to the first choice.

Proximity to the first choice significantly affected subjects' preference for tofu, dry food and chicks. Tofu was 78.58% more likely to be chosen when in proximity to the first choice. Dry food was 38.88% more likely to be chosen when in proximity to the first choice, but this difference was on the threshold of significance (P = 0.05). Chicks were 13.34% more likely to be chosen when they were not close to the first choice.

Further, feeding condition significantly affected subjects' preference for chicks. Regardless of species, subjects were 15% more likely to choose chicks when unfed than when fed (as opposed to the first choice).

The likelihood of choosing sausage was significantly higher if chicks, meat or dry food were chosen first. For complete model information and parameter estimates, see S4 File.

Food Type	Species		Feeding Condition		Species*Feeding Condition		Proximity to Choice 1 (PrC1)		PrC1*Species		PrC1*Condition	
	Wald χ^2	P	Wald χ^2	P	Wald χ^2	P	Wald χ^2	P	Wald χ^2	P	Wald χ^2	P
Chick	0.024	0.877	4.775	0.029*	2.019	0.155	6.066	0.014*	1.799	0.180	0.755	0.385
Meat	3.590	0.058	0.300	0.584	0.005	0.584	1.913	0.167	1.609	0.205	11.934	0.001*
Sausage	0.559	0.455	1.322	0.250	0.291	0.589	10.714	0.001*	1.999	0.157	1.393	0.238
Dry Food	0.971	0.324	0.017	0.897	0.056	0.813	3.858	0.05	0.092	0.761	0.402	0.526
Tofu	6.041	0.014*	5.318	0.021*	4.523	0.033*	19.681	< 0.001*	1.243	0.265	2.792	0.95

S3 Table 1: Factors predicting the likelihood of a food being chosen second.

Food Type	Choice 1: Chick		Choice 1: Meat		Choice 1: Sausage		Choice 1: Dry Food		Choice 1: Tofu	
	Wald χ^2	P	Wald χ^2	P	Wald χ^2	P	Wald χ^2	P	Wald χ^2	P
Chick	-	-	1.053	0.305	6.517	0.011	0.100	0.752	0.083	0.773
Meat	0.253	0.615	-	-	0.077	0.781	0.866	0.352	3.499	0.061
Sausage	9.731	0.002*	7.699	0.006*	-	-	11.024	0.001*	2.348	0.125
Dry Food	2.290	0.130	2.109	0.143	0.419	0.517	-	-	0.042	0.837
Tofu	0.540	0.463	0.258	0.611	0.108	0.742	0.584	0.445	-	-

S3 Table 2: Likelihood of a food being chosen second as predicted by the food chosen first (continued from S3 Table 1).
