# Supplementary material for: Food preferences of similarly raised and kept captive dogs and wolves
Source: PLoS One. 2018 Sep 20;13(9):e0203165. doi: 10.1371/journal.pone.0203165 (PMC6157812; doi:10.1371/journal.pone.0203165)
Supplement: S3 File — (DOCX) [file pone.0203165.s004.docx]

# Supplementary Information 4: Complete GEE model information for Choice 1

(Click on the arrows next to headers to expand each heading)

## Factors affecting Choice 1, Overall model

| **Model Information** | | | | | | | | | | | | | |  |  |  |  |  |
| --- | --- | --- | --- | --- | --- | --- | --- | --- | --- | --- | --- | --- | --- | --- | --- | --- | --- | --- |
| Dependent Variable | | | | | | | Choice.Food^a^ | | | | | | |  |  |  |  |  |
| Probability Distribution | | | | | | | Multinomial | | | | | | |  |  |  |  |  |
| Link Function | | | | | | | Cumulative logit | | | | | | |  |  |  |  |  |
| Subject Effect | | | 1 | | | | Animal | | | | | | |  |  |  |  |  |
| Within-Subject Effect | | | 1 | | | | Trial | | | | | | |  |  |  |  |  |
| Working Correlation Matrix Structure | | | | | | | Independent | | | | | | |  |  |  |  |  |
| a. The procedure applies the cumulative link function to the dependent variable values in ascending order. | | | | | | | | | | | | | |  |  |  |  |  |
| **Tests of Model Effects** | | | | | | | | | | |  |  |  |  |  |  |  |  |
| Source | | Type III | | | | | | | | |  |  |  |  |  |  |  |  |
|  |  | Wald Chi-Square | | | df | | | | Sig. | |  |  |  |  |  |  |  |  |
| Species | | 1.091 | | | 1 | | | | .296 | |  |  |  |  |  |  |  |  |
| **Condition** | | **4.700** | | | **1** | | | | **.030** | |  |  |  |  |  |  |  |  |
| Species * Condition | | .721 | | | 1 | | | | .396 | |  |  |  |  |  |  |  |  |
| Dependent Variable: Choice.Food  Model: (Threshold), Species, Condition, Species * Condition | | | | | | | | | | |  |  |  |  |  |  |  |  |
| **Parameter Estimates** | | | | | | | | | | | | | | | | | | |
| Parameter | | | | B | | Std. Error | | 95% Wald Confidence Interval | | | | Hypothesis Test | | | | Exp(B) | 95% Wald Confidence Interval for Exp(B) | |
|  |  |  |  |  |  |  |  | Lower | | Upper | | Wald Chi-Square | df | | Sig. |  | Lower | Upper |
| Threshold | [Choice.Food=Chick] | | | -1.130 | | .3058 | | -1.729 | | -.530 | | 13.642 | 1 | | .000 | .323 | .177 | .588 |
|  | [Choice.Food=Control] | | | -1.034 | | .2918 | | -1.606 | | -.462 | | 12.559 | 1 | | .000 | .356 | .201 | .630 |
|  | [Choice.Food=DryFood] | | | -.737 | | .2712 | | -1.269 | | -.206 | | 7.393 | 1 | | .007 | .478 | .281 | .814 |
|  | [Choice.Food=Meat] | | | .731 | | .2756 | | .191 | | 1.271 | | 7.031 | 1 | | .008 | 2.077 | 1.210 | 3.564 |
|  | [Choice.Food=Sausage] | | | 2.138 | | .3210 | | 1.509 | | 2.767 | | 44.377 | 1 | | .000 | 8.483 | 4.522 | 15.914 |
| [Species=Dog] | | | | .458 | | .3209 | | -.171 | | 1.087 | | 2.035 | 1 | | .154 | 1.581 | .843 | 2.964 |
| [Species=Wolf] | | | | 0^a^ | | . | | . | | . | | . | . | | . | 1 | . | . |
| [Condition=Fed] | | | | -.265 | | .2976 | | -.848 | | .318 | | .792 | 1 | | .374 | .767 | .428 | 1.375 |
| [Condition=Unfed] | | | | 0^a^ | | . | | . | | . | | . | . | | . | 1 | . | . |
| [Species=Dog] * [Condition=Fed] | | | | -.353 | | .4159 | | -1.168 | | .462 | | .721 | 1 | | .396 | .702 | .311 | 1.587 |
| [Species=Dog] * [Condition=Unfed] | | | | 0^a^ | | . | | . | | . | | . | . | | . | 1 | . | . |
| [Species=Wolf] * [Condition=Fed] | | | | 0^a^ | | . | | . | | . | | . | . | | . | 1 | . | . |
| [Species=Wolf] * [Condition=Unfed] | | | | 0^a^ | | . | | . | | . | | . | . | | . | 1 | . | . |
| (Scale) | | | | 1 | |  | |  | |  | |  |  | |  |  |  |  |
| Dependent Variable: Choice.Food  Model: (Threshold), Species, Condition, Species * Condition | | | | | | | | | | | | | | | | | | |
| a. Set to zero because this parameter is redundant. | | | | | | | | | | | | | | | | | | |

## Analysis for Chicks, Choice 1

| **Model Information** | | | | | | | | | | | | |  |  |  |  |  |  |
| --- | --- | --- | --- | --- | --- | --- | --- | --- | --- | --- | --- | --- | --- | --- | --- | --- | --- | --- |
| Dependent Variable | | | | | | | Chick.or.Not^a^ | | | | | |  |  |  |  |  |  |
| Probability Distribution | | | | | | | Binomial | | | | | |  |  |  |  |  |  |
| Link Function | | | | | | | Logit | | | | | |  |  |  |  |  |  |
| Subject Effect | | | 1 | | | | Animal | | | | | |  |  |  |  |  |  |
| Within-Subject Effect | | | 1 | | | | Trial | | | | | |  |  |  |  |  |  |
| Working Correlation Matrix Structure | | | | | | | Independent | | | | | |  |  |  |  |  |  |
| a. The procedure models 0 as the response, treating 1 as the reference category. | | | | | | | | | | | | |  |  |  |  |  |  |
| **Tests of Model Effects** | | | | | | | | | |  |  |  |  |  |  |  |  |  |
| Source | Type III | | | | | | | | |  |  |  |  |  |  |  |  |  |
|  | Wald Chi-Square | | | | df | | | Sig. | |  |  |  |  |  |  |  |  |  |
| (Intercept) | 32.814 | | | | 1 | | | .000 | |  |  |  |  |  |  |  |  |  |
| Species | .768 | | | | 1 | | | .381 | |  |  |  |  |  |  |  |  |  |
| **Condition** | **4.627** | | | | **1** | | | **.031** | |  |  |  |  |  |  |  |  |  |
| Species * Condition | 1.683 | | | | 1 | | | .194 | |  |  |  |  |  |  |  |  |  |
| Dependent Variable: Chick.or.Not  Model: (Intercept), Species, Condition, Species * Condition | | | | | | | | | |  |  |  |  |  |  |  |  |  |
| **Goodness of Fit^a^** | | | | | | | | | | | |  |  |  |  |  |  |  |
|  | | | | | | | | | Value | | |  |  |  |  |  |  |  |
| Quasi Likelihood under Independence Model Criterion (QIC)^b^ | | | | | | | | | 636.012 | | |  |  |  |  |  |  |  |
| Corrected Quasi Likelihood under Independence Model Criterion (QICC)^b^ | | | | | | | | | 619.407 | | |  |  |  |  |  |  |  |
| Dependent Variable: Chick.or.Not  Model: (Intercept), Species, Condition, Species * Condition^a^ | | | | | | | | | | | |  |  |  |  |  |  |  |
| a. Information criteria are in smaller-is-better form. | | | | | | | | | | | |  |  |  |  |  |  |  |
| b. Computed using the full log quasi-likelihood function. | | | | | | | | | | | |  |  |  |  |  |  |  |
| **Parameter Estimates** | | | | | | | | | | | | | | | | | | |
| Parameter | | B | | Std. Error | | 95% Wald Confidence Interval | | | | | Hypothesis Test | | | | | Exp(B) | 95% Wald Confidence Interval for Exp(B) | |
|  |  |  |  |  |  | Lower | | | Upper | | Wald Chi-Square | | | df | Sig. |  | Lower | Upper |
| (Intercept) | | 1.122 | | .4063 | | .326 | | | 1.919 | | 7.626 | | | 1 | .006 | 3.071 | 1.385 | 6.811 |
| [Species=Dog] | | .750 | | .5575 | | -.343 | | | 1.842 | | 1.808 | | | 1 | .179 | 2.116 | .710 | 6.311 |
| [Species=Wolf] | | 0^a^ | | . | | . | | | . | | . | | | . | . | 1 | . | . |
| [Condition=Fed] | | -.257 | | .3987 | | -1.039 | | | .524 | | .416 | | | 1 | .519 | .773 | .354 | 1.689 |
| [Condition=Unfed] | | 0^a^ | | . | | . | | | . | | . | | | . | . | 1 | . | . |
| [Species=Dog] * [Condition=Fed] | | -.782 | | .6025 | | -1.963 | | | .399 | | 1.683 | | | 1 | .194 | .458 | .140 | 1.491 |
| [Species=Dog] * [Condition=Unfed] | | 0^a^ | | . | | . | | | . | | . | | | . | . | 1 | . | . |
| [Species=Wolf] * [Condition=Fed] | | 0^a^ | | . | | . | | | . | | . | | | . | . | 1 | . | . |
| [Species=Wolf] * [Condition=Unfed] | | 0^a^ | | . | | . | | | . | | . | | | . | . | 1 | . | . |
| (Scale) | | 1 | |  | |  | | |  | |  | | |  |  |  |  |  |
| Dependent Variable: Chick.or.Not  Model: (Intercept), Species, Condition, Species * Condition | | | | | | | | | | | | | | | | | | |
| a. Set to zero because this parameter is redundant. | | | | | | | | | | | | | | | | | | |

## Analysis for Meat, Choice 1

| **Model Information** | | | | | | | | | | | | | |  |  |  |  |  |
| --- | --- | --- | --- | --- | --- | --- | --- | --- | --- | --- | --- | --- | --- | --- | --- | --- | --- | --- |
| Dependent Variable | | | | | | | Meat.or.Not^a^ | | | | | | |  |  |  |  |  |
| Probability Distribution | | | | | | | Binomial | | | | | | |  |  |  |  |  |
| Link Function | | | | | | | Logit | | | | | | |  |  |  |  |  |
| Subject Effect | | | 1 | | | | Animal | | | | | | |  |  |  |  |  |
| Within-Subject Effect | | | 1 | | | | Trial | | | | | | |  |  |  |  |  |
| Working Correlation Matrix Structure | | | | | | | Independent | | | | | | |  |  |  |  |  |
| a. The procedure models 0 as the response, treating 1 as the reference category. | | | | | | | | | | | | | |  |  |  |  |  |
| **Tests of Model Effects** | | | | | | | | | | | |  |  |  |  |  |  |  |
| Source | | Type III | | | | | | | | | |  |  |  |  |  |  |  |
|  |  | Wald Chi-Square | | | | df | | | Sig. | | |  |  |  |  |  |  |  |
| (Intercept) | | 21.037 | | | | 1 | | | .000 | | |  |  |  |  |  |  |  |
| Species | | .002 | | | | 1 | | | .969 | | |  |  |  |  |  |  |  |
| Condition | | 1.025 | | | | 1 | | | .311 | | |  |  |  |  |  |  |  |
| **Species * Condition** | | **5.126** | | | | **1** | | | **.024** | | |  |  |  |  |  |  |  |
| Dependent Variable: Meat.or.Not  Model: (Intercept), Species, Condition, Species * Condition | | | | | | | | | | | |  |  |  |  |  |  |  |
| **Goodness of Fit^a^** | | | | | | | | | | |  |  |  |  |  |  |  |  |
|  | | | | | | | Value | | | |  |  |  |  |  |  |  |  |
| Quasi Likelihood under Independence Model Criterion (QIC)^b^ | | | | | | | 733.329 | | | |  |  |  |  |  |  |  |  |
| Corrected Quasi Likelihood under Independence Model Criterion (QICC)^b^ | | | | | | | 722.226 | | | |  |  |  |  |  |  |  |  |
| Dependent Variable: Meat.or.Not  Model: (Intercept), Species, Condition, Species * Condition^a^ | | | | | | | | | | |  |  |  |  |  |  |  |  |
| a. Information criteria are in smaller-is-better form. | | | | | | | | | | |  |  |  |  |  |  |  |  |
| b. Computed using the full log quasi-likelihood function. | | | | | | | | | | |  |  |  |  |  |  |  |  |
| **Parameter Estimates** | | | | | | | | | | | | | | | | | | |
| Parameter | B | | | Std. Error | 95% Wald Confidence Interval | | | | | Hypothesis Test | | | | | | Exp(B) | 95% Wald Confidence Interval for Exp(B) | |
|  |  |  |  |  | Lower | | | Upper | | Wald Chi-Square | | | df | | Sig. |  | Lower | Upper |
| (Intercept) | 1.122 | | | .3335 | .468 | | | 1.776 | | 11.320 | | | 1 | | .001 | 3.071 | 1.597 | 5.905 |
| [Species=Dog] | -.633 | | | .4192 | -1.454 | | | .189 | | 2.277 | | | 1 | | .131 | .531 | .234 | 1.208 |
| [Species=Wolf] | 0^a^ | | | . | . | | | . | | . | | | . | | . | 1 | . | . |
| [Condition=Fed] | -.899 | | | .2900 | -1.467 | | | -.331 | | 9.612 | | | 1 | | .002 | .407 | .231 | .718 |
| [Condition=Unfed] | 0^a^ | | | . | . | | | . | | . | | | . | | . | 1 | . | . |
| [Species=Dog] * [Condition=Fed] | 1.242 | | | .5487 | .167 | | | 2.318 | | 5.126 | | | 1 | | .024 | 3.464 | 1.182 | 10.153 |
| [Species=Dog] * [Condition=Unfed] | 0^a^ | | | . | . | | | . | | . | | | . | | . | 1 | . | . |
| [Species=Wolf] * [Condition=Fed] | 0^a^ | | | . | . | | | . | | . | | | . | | . | 1 | . | . |
| [Species=Wolf] * [Condition=Unfed] | 0^a^ | | | . | . | | | . | | . | | | . | | . | 1 | . | . |
| (Scale) | 1 | | |  |  | | |  | |  | | |  | |  |  |  |  |
| Dependent Variable: Meat.or.Not  Model: (Intercept), Species, Condition, Species * Condition | | | | | | | | | | | | | | | | | | |
| a. Set to zero because this parameter is redundant. | | | | | | | | | | | | | | | | | | |

Estimated Marginal Means: Species * Condition

| **Estimates** | | | | | | | | | | | | | |  |  |  |  |
| --- | --- | --- | --- | --- | --- | --- | --- | --- | --- | --- | --- | --- | --- | --- | --- | --- | --- |
| Species | Condition | | | Mean | | Std. Error | | | 95% Wald Confidence Interval | | | | |  |  |  |  |
|  |  |  |  |  |  |  |  |  | Lower | | Upper | | |  |  |  |  |
| Dog | Fed | | | .70 | | .060 | | | .57 | | .80 | | |  |  |  |  |
|  | Unfed | | | .62 | | .060 | | | .50 | | .73 | | |  |  |  |  |
| Wolf | Fed | | | .56 | | .062 | | | .43 | | .67 | | |  |  |  |  |
|  | Unfed | | | .75 | | .062 | | | .62 | | .86 | | |  |  |  |  |
| **Pairwise Comparisons** | | | | | | | | | | | | | | | | |  |
| (I) Species*Condition | | | (J) Species*Condition | | | | Mean Difference (I-J) | | | Std. Error | | df | Sequential Bonferroni Sig. | | 95% Wald Confidence Interval for Difference^a^ | |  |
|  |  |  |  |  |  |  |  |  |  |  |  |  |  |  | Lower | Upper |  |
| [Species=Dog] * [Condition=Fed] | | | [Species=Dog] * [Condition=Unfed] | | | | .08 | | | .104 | | 1 | 1.000 | | -.16 | .31 |  |
|  |  |  | [Species=Wolf] * [Condition=Fed] | | | | .14 | | | .086 | | 1 | .505 | | -.08 | .36 |  |
|  |  |  | [Species=Wolf] * [Condition=Unfed] | | | | -.06 | | | .086 | | 1 | 1.000 | | -.25 | .14 |  |
| [Species=Dog] * [Condition=Unfed] | | | [Species=Dog] * [Condition=Fed] | | | | -.08 | | | .104 | | 1 | 1.000 | | -.31 | .16 |  |
|  |  |  | [Species=Wolf] * [Condition=Fed] | | | | .06 | | | .086 | | 1 | 1.000 | | -.14 | .27 |  |
|  |  |  | [Species=Wolf] * [Condition=Unfed] | | | | -.13 | | | .086 | | 1 | .505 | | -.35 | .08 |  |
| [Species=Wolf] * [Condition=Fed] | | | [Species=Dog] * [Condition=Fed] | | | | -.14 | | | .086 | | 1 | .505 | | -.36 | .08 |  |
|  |  |  | [Species=Dog] * [Condition=Unfed] | | | | -.06 | | | .086 | | 1 | 1.000 | | -.27 | .14 |  |
|  |  |  | [Species=Wolf] * [Condition=Unfed] | | | | **-.20^b^** | | | **.059** | | **1** | **.005** | | -.36 | -.04 |  |
| [Species=Wolf] * [Condition=Unfed] | | | [Species=Dog] * [Condition=Fed] | | | | .06 | | | .086 | | 1 | 1.000 | | -.14 | .25 |  |
|  |  |  | [Species=Dog] * [Condition=Unfed] | | | | .13 | | | .086 | | 1 | .505 | | -.08 | .35 |  |
|  |  |  | [Species=Wolf] * [Condition=Fed] | | | | **.20^b^** | | | **.059** | | **1** | **.005** | | .04 | .36 |  |
| Pairwise comparisons of estimated marginal means based on the original scale of dependent variable Meat.or.Not | | | | | | | | | | | | | | | | |  |
| a. Confidence interval bounds are approximate. | | | | | | | | | | | | | | | | |  |
| b. The mean difference is significant at the .05 level. | | | | | | | | | | | | | | | | |  |
| **Overall Test Results** | | | | | | | |  |  |  |  |  |  |  |  |  |  |
| Wald Chi-Square | | df | | | Sig. | | |  |  |  |  |  |  |  |  |  |  |
| 11.777 | | 3 | | | .008 | | |  |  |  |  |  |  |  |  |  |  |
| The Wald chi-square tests the effect of Species*Condition. This test is based on the linearly independent pairwise comparisons among the estimated marginal means. | | | | | | | | | | | | | | | | | |

## Analysis for Sausage, Choice 1

| **Model Information** | | | | | | | | | | | | | | |  |  |  |  |  |
| --- | --- | --- | --- | --- | --- | --- | --- | --- | --- | --- | --- | --- | --- | --- | --- | --- | --- | --- | --- |
| Dependent Variable | | | | | | | | Sausage.or.Not^a^ | | | | | | |  |  |  |  |  |
| Probability Distribution | | | | | | | | Binomial | | | | | | |  |  |  |  |  |
| Link Function | | | | | | | | Logit | | | | | | |  |  |  |  |  |
| Subject Effect | | | 1 | | | | | Animal | | | | | | |  |  |  |  |  |
| Within-Subject Effect | | | 1 | | | | | Trial | | | | | | |  |  |  |  |  |
| Working Correlation Matrix Structure | | | | | | | | Independent | | | | | | |  |  |  |  |  |
| a. The procedure models 0 as the response, treating 1 as the reference category. | | | | | | | | | | | | | | |  |  |  |  |  |
| **Tests of Model Effects** | | | | | | | | | | | | |  |  |  |  |  |  |  |
| Source | | Type III | | | | | | | | | | |  |  |  |  |  |  |  |
|  |  | Wald Chi-Square | | | | df | | | | Sig. | | |  |  |  |  |  |  |  |
| (Intercept) | | 51.400 | | | | 1 | | | | .000 | | |  |  |  |  |  |  |  |
| Species | | .241 | | | | 1 | | | | .623 | | |  |  |  |  |  |  |  |
| Condition | | 2.961 | | | | 1 | | | | .085 | | |  |  |  |  |  |  |  |
| Species * Condition | | .073 | | | | 1 | | | | .787 | | |  |  |  |  |  |  |  |
| Dependent Variable: Sausage.or.Not  Model: (Intercept), Species, Condition, Species * Condition | | | | | | | | | | | | |  |  |  |  |  |  |  |
| **Goodness of Fit^a^** | | | | | | | | | | | |  |  |  |  |  |  |  |  |
|  | | | | | | | Value | | | | |  |  |  |  |  |  |  |  |
| Quasi Likelihood under Independence Model Criterion (QIC)^b^ | | | | | | | 609.442 | | | | |  |  |  |  |  |  |  |  |
| Corrected Quasi Likelihood under Independence Model Criterion (QICC)^b^ | | | | | | | 598.425 | | | | |  |  |  |  |  |  |  |  |
| Dependent Variable: Sausage.or.Not  Model: (Intercept), Species, Condition, Species * Condition^a^ | | | | | | | | | | | |  |  |  |  |  |  |  |  |
| a. Information criteria are in smaller-is-better form. | | | | | | | | | | | |  |  |  |  |  |  |  |  |
| b. Computed using the full log quasi-likelihood function. | | | | | | | | | | | |  |  |  |  |  |  |  |  |
| **Parameter Estimates** | | | | | | | | | | | | | | | | | | | |
| Parameter | B | | | Std. Error | 95% Wald Confidence Interval | | | | | | Hypothesis Test | | | | | | Exp(B) | 95% Wald Confidence Interval for Exp(B) | |
|  |  |  |  |  | Lower | | | | Upper | | Wald Chi-Square | | | df | | Sig. |  | Lower | Upper |
| (Intercept) | 1.075 | | | .3112 | .465 | | | | 1.685 | | 11.942 | | | 1 | | .001 | 2.931 | 1.593 | 5.394 |
| [Species=Dog] | -.098 | | | .4077 | -.897 | | | | .702 | | .057 | | | 1 | | .811 | .907 | .408 | 2.017 |
| [Species=Wolf] | 0^a^ | | | . | . | | | | . | | . | | | . | | . | 1 | . | . |
| [Condition=Fed] | .561 | | | .3846 | -.193 | | | | 1.315 | | 2.127 | | | 1 | | .145 | 1.752 | .825 | 3.724 |
| [Condition=Unfed] | 0^a^ | | | . | . | | | | . | | . | | | . | | . | 1 | . | . |
| [Species=Dog] * [Condition=Fed] | -.152 | | | .5634 | -1.257 | | | | .952 | | .073 | | | 1 | | .787 | .859 | .285 | 2.590 |
| [Species=Dog] * [Condition=Unfed] | 0^a^ | | | . | . | | | | . | | . | | | . | | . | 1 | . | . |
| [Species=Wolf] * [Condition=Fed] | 0^a^ | | | . | . | | | | . | | . | | | . | | . | 1 | . | . |
| [Species=Wolf] * [Condition=Unfed] | 0^a^ | | | . | . | | | | . | | . | | | . | | . | 1 | . | . |
| (Scale) | 1 | | |  |  | | | |  | |  | | |  | |  |  |  |  |
| Dependent Variable: Sausage.or.Not  Model: (Intercept), Species, Condition, Species * Condition | | | | | | | | | | | | | | | | | | | |
| a. Set to zero because this parameter is redundant. | | | | | | | | | | | | | | | | | | | |

## Analysis for Dry Food, Choice 1

| **Model Information** | | | | | | | | | | | | | | |  |  |  |  |  |
| --- | --- | --- | --- | --- | --- | --- | --- | --- | --- | --- | --- | --- | --- | --- | --- | --- | --- | --- | --- |
| Dependent Variable | | | | | | | DryFood.or.Not^a^ | | | | | | | |  |  |  |  |  |
| Probability Distribution | | | | | | | Binomial | | | | | | | |  |  |  |  |  |
| Link Function | | | | | | | Logit | | | | | | | |  |  |  |  |  |
| Subject Effect | | 1 | | | | | Animal | | | | | | | |  |  |  |  |  |
| Within-Subject Effect | | 1 | | | | | Trial | | | | | | | |  |  |  |  |  |
| Working Correlation Matrix Structure | | | | | | | Independent | | | | | | | |  |  |  |  |  |
| a. The procedure models 0 as the response, treating 1 as the reference category. | | | | | | | | | | | | | | |  |  |  |  |  |
| **Tests of Model Effects** | | | | | | | | | | | |  |  |  |  |  |  |  |  |
| Source | Type III | | | | | | | | | | |  |  |  |  |  |  |  |  |
|  | Wald Chi-Square | | | | df | | | | Sig. | | |  |  |  |  |  |  |  |  |
| (Intercept) | 140.193 | | | | 1 | | | | .000 | | |  |  |  |  |  |  |  |  |
| Species | .001 | | | | 1 | | | | .979 | | |  |  |  |  |  |  |  |  |
| Condition | 2.573 | | | | 1 | | | | .109 | | |  |  |  |  |  |  |  |  |
| Species * Condition | 1.905 | | | | 1 | | | | .168 | | |  |  |  |  |  |  |  |  |
| Dependent Variable: DryFood.or.Not  Model: (Intercept), Species, Condition, Species * Condition | | | | | | | | | | | |  |  |  |  |  |  |  |  |
| **Goodness of Fit^a^** | | | | | | | | | | |  |  |  |  |  |  |  |  |  |
|  | | | | | | | | Value | | |  |  |  |  |  |  |  |  |  |
| Quasi Likelihood under Independence Model Criterion (QIC)^b^ | | | | | | | | 265.105 | | |  |  |  |  |  |  |  |  |  |
| Corrected Quasi Likelihood under Independence Model Criterion (QICC)^b^ | | | | | | | | 258.700 | | |  |  |  |  |  |  |  |  |  |
| Dependent Variable: DryFood.or.Not  Model: (Intercept), Species, Condition, Species * Condition^a^ | | | | | | | | | | |  |  |  |  |  |  |  |  |  |
| a. Information criteria are in smaller-is-better form. | | | | | | | | | | |  |  |  |  |  |  |  |  |  |
| b. Computed using the full log quasi-likelihood function. | | | | | | | | | | |  |  |  |  |  |  |  |  |  |
| **Parameter Estimates** | | | | | | | | | | | | | | | | | | | |
| Parameter | | | B | Std. Error | | 95% Wald Confidence Interval | | | | | | | Hypothesis Test | | | | Exp(B) | 95% Wald Confidence Interval for Exp(B) | |
|  |  |  |  |  |  | Lower | | | | Upper | | | Wald Chi-Square | df | | Sig. |  | Lower | Upper |
| (Intercept) | | | 2.140 | .5015 | | 1.157 | | | | 3.123 | | | 18.211 | 1 | | .000 | 8.500 | 3.181 | 22.714 |
| [Species=Dog] | | | .611 | .7035 | | -.767 | | | | 1.990 | | | .756 | 1 | | .385 | 1.843 | .464 | 7.317 |
| [Species=Wolf] | | | 0^a^ | . | | . | | | | . | | | . | . | | . | 1 | . | . |
| [Condition=Fed] | | | 1.349 | .5142 | | .341 | | | | 2.357 | | | 6.882 | 1 | | .009 | 3.853 | 1.406 | 10.555 |
| [Condition=Unfed] | | | 0^a^ | . | | . | | | | . | | | . | . | | . | 1 | . | . |
| [Species=Dog] * [Condition=Fed] | | | -1.248 | .9040 | | -3.020 | | | | .524 | | | 1.905 | 1 | | .168 | .287 | .049 | 1.689 |
| [Species=Dog] * [Condition=Unfed] | | | 0^a^ | . | | . | | | | . | | | . | . | | . | 1 | . | . |
| [Species=Wolf] * [Condition=Fed] | | | 0^a^ | . | | . | | | | . | | | . | . | | . | 1 | . | . |
| [Species=Wolf] * [Condition=Unfed] | | | 0^a^ | . | | . | | | | . | | | . | . | | . | 1 | . | . |
| (Scale) | | | 1 |  | |  | | | |  | | |  |  | |  |  |  |  |
| Dependent Variable: DryFood.or.Not  Model: (Intercept), Species, Condition, Species * Condition | | | | | | | | | | | | | | | | | | | |
| a. Set to zero because this parameter is redundant. | | | | | | | | | | | | | | | | | | | |

## Analysis for Tofu, Choice 1

| **Model Information** | | | | | | |
| --- | --- | --- | --- | --- | --- | --- |
| Dependent Variable | | | | Tofu.or.Not^a^ | | |
| Probability Distribution | | | | Binomial | | |
| Link Function | | | | Logit | | |
| Subject Effect | | 1 | | Animal | | |
| Within-Subject Effect | | 1 | | Trial | | |
| Working Correlation Matrix Structure | | | | Independent | | |
| a. The procedure models 0 as the response, treating 1 as the reference category. | | | | | | |
| **Tests of Model Effects** | | | | | |  |
| Source | Type III | | | | |  |
|  | Wald Chi-Square | | df | | Sig. |  |
| (Intercept) | 116.726 | | 1 | | .000 |  |
| Species | 1.114 | | 1 | | .291 |  |
| Condition | .949 | | 1 | | .330 |  |
| Species * Condition | .191 | | 1 | | .662 |  |
| Dependent Variable: Tofu.or.Not  Model: (Intercept), Species, Condition, Species * Condition | | | | | |  |

| **Goodness of Fit^a^** | | | | | | | | | | | | | | | |  |  |  |  |  |  |  |  |  |  |  |  |  |  |  |  |
| --- | --- | --- | --- | --- | --- | --- | --- | --- | --- | --- | --- | --- | --- | --- | --- | --- | --- | --- | --- | --- | --- | --- | --- | --- | --- | --- | --- | --- | --- | --- | --- |
|  | | | | | | | | | | Value | | | | | |  |  |  |  |  |  |  |  |  |  |  |  |  |  |  |  |
| Quasi Likelihood under Independence Model Criterion (QIC)^b^ | | | | | | | | | | 403.218 | | | | | |  |  |  |  |  |  |  |  |  |  |  |  |  |  |  |  |
| Corrected Quasi Likelihood under Independence Model Criterion (QICC)^b^ | | | | | | | | | | 394.391 | | | | | |  |  |  |  |  |  |  |  |  |  |  |  |  |  |  |  |
| Dependent Variable: Tofu.or.Not  Model: (Intercept), Species, Condition, Species * Condition^a^ | | | | | | | | | | | | | | | |  |  |  |  |  |  |  |  |  |  |  |  |  |  |  |  |
| a. Information criteria are in smaller-is-better form. | | | | | | | | | | | | | | | |  |  |  |  |  |  |  |  |  |  |  |  |  |  |  |  |
| b. Computed using the full log quasi-likelihood function. | | | | | | | | | | | | | | | |  |  |  |  |  |  |  |  |  |  |  |  |  |  |  |  |
| **Parameter Estimates** | | | | | | | | | | | | | | | | | | | | | | | | | | | | | | | |
| Parameter | | B | | Std. Error | | | 95% Wald Confidence Interval | | | | | | | Hypothesis Test | | | | | | | | | | | Exp(B) | | 95% Wald Confidence Interval for Exp(B) | | | | |
|  |  |  |  |  |  |  | Lower | | | | Upper | | | Wald Chi-Square | | | | | df | | | | Sig. | |  |  | Lower | | Upper | | |
| (Intercept) | | 2.050 | | .4734 | | | 1.122 | | | | 2.978 | | | 18.752 | | | | | 1 | | | | .000 | | 7.769 | | 3.072 | | 19.650 | | |
| [Species=Dog] | | -.235 | | .5957 | | | -1.402 | | | | .933 | | | .155 | | | | | 1 | | | | .693 | | .791 | | .246 | | 2.541 | | |
| [Species=Wolf] | | 0^a^ | | . | | | . | | | | . | | | . | | | | | . | | | | . | | 1 | | . | | . | | |
| [Condition=Fed] | | .589 | | .6171 | | | -.621 | | | | 1.798 | | | .911 | | | | | 1 | | | | .340 | | 1.802 | | .538 | | 6.040 | | |
| [Condition=Unfed] | | 0^a^ | | . | | | . | | | | . | | | . | | | | | . | | | | . | | 1 | | . | | . | | |
| [Species=Dog] * [Condition=Fed] | | -.365 | | .8342 | | | -2.000 | | | | 1.270 | | | .191 | | | | | 1 | | | | .662 | | .694 | | .135 | | 3.561 | | |
| [Species=Dog] * [Condition=Unfed] | | 0^a^ | | . | | | . | | | | . | | | . | | | | | . | | | | . | | 1 | | . | | . | | |
| [Species=Wolf] * [Condition=Fed] | | 0^a^ | | . | | | . | | | | . | | | . | | | | | . | | | | . | | 1 | | . | | . | | |
| [Species=Wolf] * [Condition=Unfed] | | 0^a^ | | . | | | . | | | | . | | | . | | | | | . | | | | . | | 1 | | . | | . | | |
| (Scale) | | 1 | |  | | |  | | | |  | | |  | | | | |  | | | |  | |  | |  | |  | | |
| Dependent Variable: Tofu.or.Not  Model: (Intercept), Species, Condition, Species * Condition | | | | | | | | | | | | | | | | | | | | | | | | | | | | | | | |
| a. Set to zero because this parameter is redundant. | | | | | | | | | | | | | | | | | | | | | | | | | | | | | | | |
| **Model Information** | | | | | | | | | | | | | | | | | | | |  |  |  |  |  |  |  |  |  |  |  |  |
| Dependent Variable | | | | | | | | Choice.2^a^ | | | | | | | | | | | |  |  |  |  |  |  |  |  |  |  |  |  |
| Probability Distribution | | | | | | | | Multinomial | | | | | | | | | | | |  |  |  |  |  |  |  |  |  |  |  |  |
| Link Function | | | | | | | | Cumulative logit | | | | | | | | | | | |  |  |  |  |  |  |  |  |  |  |  |  |
| Subject Effect | | | 1 | | | | | Animal | | | | | | | | | | | |  |  |  |  |  |  |  |  |  |  |  |  |
| Within-Subject Effect | | | 1 | | | | | Trial | | | | | | | | | | | |  |  |  |  |  |  |  |  |  |  |  |  |
| Working Correlation Matrix Structure | | | | | | | | Independent | | | | | | | | | | | |  |  |  |  |  |  |  |  |  |  |  |  |
| a. The procedure applies the cumulative link function to the dependent variable values in ascending order. | | | | | | | | | | | | | | | | | | | |  |  |  |  |  |  |  |  |  |  |  |  |
| **Tests of Model Effects** | | | | | | | | | | | | | | | | | | | | | |  |  |  |  |  |  |  |  |  |  |
| Source | | | | | Type III | | | | | | | | | | | | | | | | |  |  |  |  |  |  |  |  |  |  |
|  |  |  |  |  | Wald Chi-Square | | | | | | | | df | | | | Sig. | | | | |  |  |  |  |  |  |  |  |  |  |
| Species | | | | | .231 | | | | | | | | 1 | | | | .631 | | | | |  |  |  |  |  |  |  |  |  |  |
| Condition | | | | | 3.094 | | | | | | | | 1 | | | | .079 | | | | |  |  |  |  |  |  |  |  |  |  |
| Proximity.to.Choice.1 | | | | | 2.254 | | | | | | | | 1 | | | | .133 | | | | |  |  |  |  |  |  |  |  |  |  |
| Species * Condition | | | | | 1.926 | | | | | | | | 1 | | | | .165 | | | | |  |  |  |  |  |  |  |  |  |  |
| Species * Proximity.to.Choice.1 | | | | | 2.001 | | | | | | | | 1 | | | | .157 | | | | |  |  |  |  |  |  |  |  |  |  |
| Condition * Proximity.to.Choice.1 | | | | | .006 | | | | | | | | 1 | | | | .936 | | | | |  |  |  |  |  |  |  |  |  |  |
| C1.Chick.or.Not | | | | | 2.557 | | | | | | | | 1 | | | | .110 | | | | |  |  |  |  |  |  |  |  |  |  |
| C1.Meat.or.Not | | | | | 2.920 | | | | | | | | 1 | | | | .087 | | | | |  |  |  |  |  |  |  |  |  |  |
| **C1.Sausage.or.Not** | | | | | **5.486** | | | | | | | | **1** | | | | **.019** | | | | |  |  |  |  |  |  |  |  |  |  |
| C1.DryFood.or.Not | | | | | 1.892 | | | | | | | | 1 | | | | .169 | | | | |  |  |  |  |  |  |  |  |  |  |
| C1.Tofu.or.Not | | | | | 3.762 | | | | | | | | 1 | | | | .052 | | | | |  |  |  |  |  |  |  |  |  |  |
| Dependent Variable: Choice.2  Model: (Threshold), Species, Condition, Proximity.to.Choice.1, Species * Condition, Species * Proximity.to.Choice.1, Condition * Proximity.to.Choice.1, C1.Chick.or.Not, C1.Meat.or.Not, C1.Sausage.or.Not, C1.DryFood.or.Not, C1.Tofu.or.Not | | | | | | | | | | | | | | | | | | | | | |  |  |  |  |  |  |  |  |  |  |
| **Parameter Estimates** | | | | | | | | | | | | | | | | | | | | | | | | | | | | | | |  |
| Parameter | | | | | | B | | | Std. Error | | | 95% Wald Confidence Interval | | | | | | Hypothesis Test | | | | | | | | Exp(B) | | 95% Wald Confidence Interval for Exp(B) | | |  |
|  |  |  |  |  |  |  |  |  |  |  |  | Lower | | | Upper | | | Wald Chi-Square | | | df | | | Sig. | |  |  | Lower | | Upper |  |
| Threshold | [Choice.2=Chick] | | | | | -2.014 | | | .6308 | | | -3.250 | | | -.778 | | | 10.193 | | | 1 | | | .001 | | .133 | | .039 | | .460 |  |
|  | [Choice.2=Control] | | | | | -1.776 | | | .6456 | | | -3.041 | | | -.510 | | | 7.564 | | | 1 | | | .006 | | .169 | | .048 | | .600 |  |
|  | [Choice.2=DryFood] | | | | | -1.165 | | | .6463 | | | -2.432 | | | .102 | | | 3.250 | | | 1 | | | .071 | | .312 | | .088 | | 1.107 |  |
|  | [Choice.2=Meat] | | | | | -.262 | | | .6650 | | | -1.565 | | | 1.041 | | | .155 | | | 1 | | | .694 | | .770 | | .209 | | 2.833 |  |
|  | [Choice.2=Nil] | | | | | -.170 | | | .6611 | | | -1.466 | | | 1.125 | | | .066 | | | 1 | | | .797 | | .843 | | .231 | | 3.082 |  |
|  | [Choice.2=Sausage] | | | | | 1.125 | | | .6880 | | | -.223 | | | 2.474 | | | 2.674 | | | 1 | | | .102 | | 3.080 | | .800 | | 11.864 |  |
| [Species=Dog] | | | | | | .782 | | | .4220 | | | -.046 | | | 1.609 | | | 3.430 | | | 1 | | | .064 | | 2.185 | | .955 | | 4.997 |  |
| [Species=Wolf] | | | | | | 0^a^ | | | . | | | . | | | . | | | . | | | . | | | . | | 1 | | . | | . |  |
| **[Condition=Fed]** | | | | | | **.822** | | | **.3867** | | | **.064** | | | **1.580** | | | **4.522** | | | **1** | | | **.033** | | **2.276** | | **1.067** | | **4.856** |  |
| [Condition=Unfed] | | | | | | 0^a^ | | | . | | | . | | | . | | | . | | | . | | | . | | 1 | | . | | . |  |
| [Proximity.to.Choice.1=No] | | | | | | .003 | | | .2995 | | | -.584 | | | .590 | | | .000 | | | 1 | | | .992 | | 1.003 | | .558 | | 1.804 |  |
| [Proximity.to.Choice.1=Yes] | | | | | | 0^a^ | | | . | | | . | | | . | | | . | | | . | | | . | | 1 | | . | | . |  |
| [Species=Dog] * [Condition=Fed] | | | | | | -.708 | | | .5098 | | | -1.707 | | | .292 | | | 1.926 | | | 1 | | | .165 | | .493 | | .181 | | 1.339 |  |
| [Species=Dog] * [Condition=Unfed] | | | | | | 0^a^ | | | . | | | . | | | . | | | . | | | . | | | . | | 1 | | . | | . |  |
| [Species=Wolf] * [Condition=Fed] | | | | | | 0^a^ | | | . | | | . | | | . | | | . | | | . | | | . | | 1 | | . | | . |  |
| [Species=Wolf] * [Condition=Unfed] | | | | | | 0^a^ | | | . | | | . | | | . | | | . | | | . | | | . | | 1 | | . | | . |  |
| [Species=Dog] * [Proximity.to.Choice.1=No] | | | | | | -.639 | | | .4518 | | | -1.525 | | | .246 | | | 2.001 | | | 1 | | | .157 | | .528 | | .218 | | 1.279 |  |
| [Species=Dog] * [Proximity.to.Choice.1=Yes] | | | | | | 0^a^ | | | . | | | . | | | . | | | . | | | . | | | . | | 1 | | . | | . |  |
| [Species=Wolf] * [Proximity.to.Choice.1=No] | | | | | | 0^a^ | | | . | | | . | | | . | | | . | | | . | | | . | | 1 | | . | | . |  |
| [Species=Wolf] * [Proximity.to.Choice.1=Yes] | | | | | | 0^a^ | | | . | | | . | | | . | | | . | | | . | | | . | | 1 | | . | | . |  |
| [Condition=Fed] * [Proximity.to.Choice.1=No] | | | | | | -.033 | | | .4085 | | | -.833 | | | .768 | | | .006 | | | 1 | | | .936 | | .968 | | .435 | | 2.155 |  |
| [Condition=Fed] * [Proximity.to.Choice.1=Yes] | | | | | | 0^a^ | | | . | | | . | | | . | | | . | | | . | | | . | | 1 | | . | | . |  |
| [Condition=Unfed] * [Proximity.to.Choice.1=No] | | | | | | 0^a^ | | | . | | | . | | | . | | | . | | | . | | | . | | 1 | | . | | . |  |
| [Condition=Unfed] * [Proximity.to.Choice.1=Yes] | | | | | | 0^a^ | | | . | | | . | | | . | | | . | | | . | | | . | | 1 | | . | | . |  |
| C1.Chick.or.Not | | | | | | -1.044 | | | .6529 | | | -2.324 | | | .236 | | | 2.557 | | | 1 | | | .110 | | .352 | | .098 | | 1.266 |  |
| C1.Meat.or.Not | | | | | | -1.147 | | | .6715 | | | -2.464 | | | .169 | | | 2.920 | | | 1 | | | .087 | | .317 | | .085 | | 1.184 |  |
| **C1.Sausage.or.Not** | | | | | | **-1.526** | | | **.6513** | | | **-2.802** | | | **-.249** | | | **5.486** | | | **1** | | | **.019** | | **.218** | | **.061** | | **.780** |  |
| C1.DryFood.or.Not | | | | | | -.906 | | | .6587 | | | -2.197 | | | .385 | | | 1.892 | | | 1 | | | .169 | | .404 | | .111 | | 1.469 |  |
| C1.Tofu.or.Not | | | | | | -1.026 | | | .5288 | | | -2.062 | | | .011 | | | 3.762 | | | 1 | | | .052 | | .359 | | .127 | | 1.011 |  |
| (Scale) | | | | | | 1 | | |  | | |  | | |  | | |  | | |  | | |  | |  | |  | |  |  |
| Dependent Variable: Choice.2  Model: (Threshold), Species, Condition, Proximity.to.Choice.1, Species * Condition, Species * Proximity.to.Choice.1, Condition * Proximity.to.Choice.1, C1.Chick.or.Not, C1.Meat.or.Not, C1.Sausage.or.Not, C1.DryFood.or.Not, C1.Tofu.or.Not | | | | | | | | | | | | | | | | | | | | | | | | | | | | | | |  |
| a. Set to zero because this parameter is redundant. | | | | | | | | | | | | | | | | | | | | | | | | | | | | | | |  |
