# Supplementary material for: Food preferences of similarly raised and kept captive dogs and wolves
Source: PLoS One. 2018 Sep 20;13(9):e0203165. doi: 10.1371/journal.pone.0203165 (PMC6157812; doi:10.1371/journal.pone.0203165)
Supplement: S4 File — (DOCX) [file pone.0203165.s005.docx]

# Supplementary Information 5: Complete GEE model information for Choice 2

(Click on the arrows next to headers to expand each heading)

## Factors affecting Choice 2, Overall model

| **Model Information** | | | | | | | | | | | |  |  |  |  |  |  |  |
| --- | --- | --- | --- | --- | --- | --- | --- | --- | --- | --- | --- | --- | --- | --- | --- | --- | --- | --- |
| Dependent Variable | | | | | Choice.2^a^ | | | | | | |  |  |  |  |  |  |  |
| Probability Distribution | | | | | Multinomial | | | | | | |  |  |  |  |  |  |  |
| Link Function | | | | | Cumulative logit | | | | | | |  |  |  |  |  |  |  |
| Subject Effect | | 1 | | | Animal | | | | | | |  |  |  |  |  |  |  |
| Within-Subject Effect | | 1 | | | Trial | | | | | | |  |  |  |  |  |  |  |
| Working Correlation Matrix Structure | | | | | Independent | | | | | | |  |  |  |  |  |  |  |
| a. The procedure applies the cumulative link function to the dependent variable values in ascending order. | | | | | | | | | | | |  |  |  |  |  |  |  |
| **Tests of Model Effects** | | | | | | | | | | | | | |  |  |  |  |  |
| Source | | | Type III | | | | | | | | | | |  |  |  |  |  |
|  |  |  | Wald Chi-Square | | | | | df | | Sig. | | | |  |  |  |  |  |
| Species | | | .231 | | | | | 1 | | .631 | | | |  |  |  |  |  |
| Condition | | | 3.094 | | | | | 1 | | .079 | | | |  |  |  |  |  |
| Proximity.to.Choice.1 | | | 2.254 | | | | | 1 | | .133 | | | |  |  |  |  |  |
| Species * Condition | | | 1.926 | | | | | 1 | | .165 | | | |  |  |  |  |  |
| Species * Proximity.to.Choice.1 | | | 2.001 | | | | | 1 | | .157 | | | |  |  |  |  |  |
| Condition * Proximity.to.Choice.1 | | | .006 | | | | | 1 | | .936 | | | |  |  |  |  |  |
| C1.Chick.or.Not | | | 2.557 | | | | | 1 | | .110 | | | |  |  |  |  |  |
| C1.Meat.or.Not | | | 2.920 | | | | | 1 | | .087 | | | |  |  |  |  |  |
| **C1.Sausage.or.Not** | | | **5.486** | | | | | **1** | | **.019** | | | |  |  |  |  |  |
| C1.DryFood.or.Not | | | 1.892 | | | | | 1 | | .169 | | | |  |  |  |  |  |
| C1.Tofu.or.Not | | | 3.762 | | | | | 1 | | .052 | | | |  |  |  |  |  |
| Dependent Variable: Choice.2  Model: (Threshold), Species, Condition, Proximity.to.Choice.1, Species * Condition, Species * Proximity.to.Choice.1, Condition * Proximity.to.Choice.1, C1.Chick.or.Not, C1.Meat.or.Not, C1.Sausage.or.Not, C1.DryFood.or.Not, C1.Tofu.or.Not | | | | | | | | | | | | | |  |  |  |  |  |
| **Parameter Estimates** | | | | | | | | | | | | | | | | | | |
| Parameter | | | | B | | Std. Error | 95% Wald Confidence Interval | | | | Hypothesis Test | | | | | Exp(B) | 95% Wald Confidence Interval for Exp(B) | |
|  |  |  |  |  |  |  | Lower | | Upper | | Wald Chi-Square | | df | | Sig. |  | Lower | Upper |
| Threshold | [Choice.2=Chick] | | | -2.014 | | .6308 | -3.250 | | -.778 | | 10.193 | | 1 | | .001 | .133 | .039 | .460 |
|  | [Choice.2=Control] | | | -1.776 | | .6456 | -3.041 | | -.510 | | 7.564 | | 1 | | .006 | .169 | .048 | .600 |
|  | [Choice.2=DryFood] | | | -1.165 | | .6463 | -2.432 | | .102 | | 3.250 | | 1 | | .071 | .312 | .088 | 1.107 |
|  | [Choice.2=Meat] | | | -.262 | | .6650 | -1.565 | | 1.041 | | .155 | | 1 | | .694 | .770 | .209 | 2.833 |
|  | [Choice.2=Nil] | | | -.170 | | .6611 | -1.466 | | 1.125 | | .066 | | 1 | | .797 | .843 | .231 | 3.082 |
|  | [Choice.2=Sausage] | | | 1.125 | | .6880 | -.223 | | 2.474 | | 2.674 | | 1 | | .102 | 3.080 | .800 | 11.864 |
| [Species=Dog] | | | | .782 | | .4220 | -.046 | | 1.609 | | 3.430 | | 1 | | .064 | 2.185 | .955 | 4.997 |
| [Species=Wolf] | | | | 0^a^ | | . | . | | . | | . | | . | | . | 1 | . | . |
| **[Condition=Fed]** | | | | **.822** | | **.3867** | **.064** | | **1.580** | | **4.522** | | **1** | | **.033** | **2.276** | **1.067** | **4.856** |
| [Condition=Unfed] | | | | 0^a^ | | . | . | | . | | . | | . | | . | 1 | . | . |
| [Proximity.to.Choice.1=No] | | | | .003 | | .2995 | -.584 | | .590 | | .000 | | 1 | | .992 | 1.003 | .558 | 1.804 |
| [Proximity.to.Choice.1=Yes] | | | | 0^a^ | | . | . | | . | | . | | . | | . | 1 | . | . |
| [Species=Dog] * [Condition=Fed] | | | | -.708 | | .5098 | -1.707 | | .292 | | 1.926 | | 1 | | .165 | .493 | .181 | 1.339 |
| [Species=Dog] * [Condition=Unfed] | | | | 0^a^ | | . | . | | . | | . | | . | | . | 1 | . | . |
| [Species=Wolf] * [Condition=Fed] | | | | 0^a^ | | . | . | | . | | . | | . | | . | 1 | . | . |
| [Species=Wolf] * [Condition=Unfed] | | | | 0^a^ | | . | . | | . | | . | | . | | . | 1 | . | . |
| [Species=Dog] * [Proximity.to.Choice.1=No] | | | | -.639 | | .4518 | -1.525 | | .246 | | 2.001 | | 1 | | .157 | .528 | .218 | 1.279 |
| [Species=Dog] * [Proximity.to.Choice.1=Yes] | | | | 0^a^ | | . | . | | . | | . | | . | | . | 1 | . | . |
| [Species=Wolf] * [Proximity.to.Choice.1=No] | | | | 0^a^ | | . | . | | . | | . | | . | | . | 1 | . | . |
| [Species=Wolf] * [Proximity.to.Choice.1=Yes] | | | | 0^a^ | | . | . | | . | | . | | . | | . | 1 | . | . |
| [Condition=Fed] * [Proximity.to.Choice.1=No] | | | | -.033 | | .4085 | -.833 | | .768 | | .006 | | 1 | | .936 | .968 | .435 | 2.155 |
| [Condition=Fed] * [Proximity.to.Choice.1=Yes] | | | | 0^a^ | | . | . | | . | | . | | . | | . | 1 | . | . |
| [Condition=Unfed] * [Proximity.to.Choice.1=No] | | | | 0^a^ | | . | . | | . | | . | | . | | . | 1 | . | . |
| [Condition=Unfed] * [Proximity.to.Choice.1=Yes] | | | | 0^a^ | | . | . | | . | | . | | . | | . | 1 | . | . |
| C1.Chick.or.Not | | | | -1.044 | | .6529 | -2.324 | | .236 | | 2.557 | | 1 | | .110 | .352 | .098 | 1.266 |
| C1.Meat.or.Not | | | | -1.147 | | .6715 | -2.464 | | .169 | | 2.920 | | 1 | | .087 | .317 | .085 | 1.184 |
| **C1.Sausage.or.Not** | | | | **-1.526** | | **.6513** | **-2.802** | | **-.249** | | **5.486** | | **1** | | **.019** | **.218** | **.061** | **.780** |
| C1.DryFood.or.Not | | | | -.906 | | .6587 | -2.197 | | .385 | | 1.892 | | 1 | | .169 | .404 | .111 | 1.469 |
| C1.Tofu.or.Not | | | | -1.026 | | .5288 | -2.062 | | .011 | | 3.762 | | 1 | | .052 | .359 | .127 | 1.011 |
| (Scale) | | | | 1 | |  |  | |  | |  | |  | |  |  |  |  |
| Dependent Variable: Choice.2  Model: (Threshold), Species, Condition, Proximity.to.Choice.1, Species * Condition, Species * Proximity.to.Choice.1, Condition * Proximity.to.Choice.1, C1.Chick.or.Not, C1.Meat.or.Not, C1.Sausage.or.Not, C1.DryFood.or.Not, C1.Tofu.or.Not | | | | | | | | | | | | | | | | | | |
| a. Set to zero because this parameter is redundant. | | | | | | | | | | | | | | | | | | |

## Analysis for Chicks, Choice 2

| **Model Information** | | | | | | | | | | | | |  |  |  |  |  |  |
| --- | --- | --- | --- | --- | --- | --- | --- | --- | --- | --- | --- | --- | --- | --- | --- | --- | --- | --- |
| Dependent Variable | | | | | C2.Chick.or.Not^a^ | | | | | | | |  |  |  |  |  |  |
| Probability Distribution | | | | | Binomial | | | | | | | |  |  |  |  |  |  |
| Link Function | | | | | Logit | | | | | | | |  |  |  |  |  |  |
| Subject Effect | 1 | | | | Animal | | | | | | | |  |  |  |  |  |  |
| Within-Subject Effect | 1 | | | | Trial | | | | | | | |  |  |  |  |  |  |
| Working Correlation Matrix Structure | | | | | Independent | | | | | | | |  |  |  |  |  |  |
| a. The procedure models 0 as the response, treating 1 as the reference category. | | | | | | | | | | | | |  |  |  |  |  |  |
| **Tests of Model Effects** | | | | | | | | | | | | | |  |  |  |  |  |
| Source | | | Type III | | | | | | | | | | |  |  |  |  |  |
|  |  |  | Wald Chi-Square | | | df | | | | Sig. | | | |  |  |  |  |  |
| (Intercept) | | | 25.373 | | | 1 | | | | .000 | | | |  |  |  |  |  |
| Species | | | .024 | | | 1 | | | | .877 | | | |  |  |  |  |  |
| **Condition** | | | **4.775** | | | **1** | | | | **.029** | | | |  |  |  |  |  |
| Species * Condition | | | 2.019 | | | 1 | | | | .155 | | | |  |  |  |  |  |
| **Proximity.to.Choice.1** | | | **6.066** | | | **1** | | | | **.014** | | | |  |  |  |  |  |
| Species * Proximity.to.Choice.1 | | | 1.799 | | | 1 | | | | .180 | | | |  |  |  |  |  |
| Condition * Proximity.to.Choice.1 | | | .755 | | | 1 | | | | .385 | | | |  |  |  |  |  |
| C1.Meat.or.Not | | | 1.053 | | | 1 | | | | .305 | | | |  |  |  |  |  |
| **C1.Sausage.or.Not** | | | **6.517** | | | **1** | | | | **.011** | | | |  |  |  |  |  |
| C1.DryFood.or.Not | | | .100 | | | 1 | | | | .752 | | | |  |  |  |  |  |
| C1.Tofu.or.Not | | | .083 | | | 1 | | | | .773 | | | |  |  |  |  |  |
| Dependent Variable: C2.Chick.or.Not  Model: (Intercept), Species, Condition, Species * Condition, Proximity.to.Choice.1, Species * Proximity.to.Choice.1, Condition * Proximity.to.Choice.1, C1.Meat.or.Not, C1.Sausage.or.Not, C1.DryFood.or.Not, C1.Tofu.or.Not | | | | | | | | | | | | | |  |  |  |  |  |
| **Goodness of Fit^a^** | | | | | | | | | | | |  |  |  |  |  |  |  |
|  | | | | | | | | Value | | | |  |  |  |  |  |  |  |
| Quasi Likelihood under Independence Model Criterion (QIC)^b^ | | | | | | | | 588.161 | | | |  |  |  |  |  |  |  |
| Corrected Quasi Likelihood under Independence Model Criterion (QICC)^b^ | | | | | | | | 571.113 | | | |  |  |  |  |  |  |  |
| Dependent Variable: C2.Chick.or.Not  Model: (Intercept), Species, Condition, Species * Condition, Proximity.to.Choice.1, Species * Proximity.to.Choice.1, Condition * Proximity.to.Choice.1, C1.Meat.or.Not, C1.Sausage.or.Not, C1.DryFood.or.Not, C1.Tofu.or.Not^a^ | | | | | | | | | | | |  |  |  |  |  |  |  |
| a. Information criteria are in smaller-is-better form. | | | | | | | | | | | |  |  |  |  |  |  |  |
| b. Computed using the full log quasi-likelihood function. | | | | | | | | | | | |  |  |  |  |  |  |  |
| **Parameter Estimates** | | | | | | | | | | | | | | | | | | |
| Parameter | | B | | Std. Error | 95% Wald Confidence Interval | | | | Hypothesis Test | | | | | | | Exp(B) | 95% Wald Confidence Interval for Exp(B) | |
|  |  |  |  |  | Lower | | Upper | | Wald Chi-Square | | df | | | | Sig. |  | Lower | Upper |
| (Intercept) | | 1.201 | | .3648 | .486 | | 1.916 | | 10.839 | | 1 | | | | .001 | 3.324 | 1.626 | 6.794 |
| [Species=Dog] | | .787 | | .5209 | -.234 | | 1.808 | | 2.283 | | 1 | | | | .131 | 2.197 | .792 | 6.099 |
| [Species=Wolf] | | 0^a^ | | . | . | | . | | . | | . | | | | . | 1 | . | . |
| [Condition=Fed] | | 1.324 | | .4901 | .364 | | 2.285 | | 7.300 | | 1 | | | | .007 | 3.759 | 1.439 | 9.824 |
| [Condition=Unfed] | | 0^a^ | | . | . | | . | | . | | . | | | | . | 1 | . | . |
| [Species=Dog] * [Condition=Fed] | | -.788 | | .5545 | -1.875 | | .299 | | 2.019 | | 1 | | | | .155 | .455 | .153 | 1.348 |
| [Species=Dog] * [Condition=Unfed] | | 0^a^ | | . | . | | . | | . | | . | | | | . | 1 | . | . |
| [Species=Wolf] * [Condition=Fed] | | 0^a^ | | . | . | | . | | . | | . | | | | . | 1 | . | . |
| [Species=Wolf] * [Condition=Unfed] | | 0^a^ | | . | . | | . | | . | | . | | | | . | 1 | . | . |
| [Proximity.to.Choice.1=No] | | .009 | | .3596 | -.696 | | .713 | | .001 | | 1 | | | | .981 | 1.009 | .498 | 2.041 |
| [Proximity.to.Choice.1=Yes] | | 0^a^ | | . | . | | . | | . | | . | | | | . | 1 | . | . |
| [Species=Dog] * [Proximity.to.Choice.1=No] | | -.671 | | .4999 | -1.650 | | .309 | | 1.799 | | 1 | | | | .180 | .511 | .192 | 1.362 |
| [Species=Dog] * [Proximity.to.Choice.1=Yes] | | 0^a^ | | . | . | | . | | . | | . | | | | . | 1 | . | . |
| [Species=Wolf] * [Proximity.to.Choice.1=No] | | 0^a^ | | . | . | | . | | . | | . | | | | . | 1 | . | . |
| [Species=Wolf] * [Proximity.to.Choice.1=Yes] | | 0^a^ | | . | . | | . | | . | | . | | | | . | 1 | . | . |
| [Condition=Fed] * [Proximity.to.Choice.1=No] | | -.556 | | .6404 | -1.811 | | .699 | | .755 | | 1 | | | | .385 | .573 | .163 | 2.011 |
| [Condition=Fed] * [Proximity.to.Choice.1=Yes] | | 0^a^ | | . | . | | . | | . | | . | | | | . | 1 | . | . |
| [Condition=Unfed] * [Proximity.to.Choice.1=No] | | 0^a^ | | . | . | | . | | . | | . | | | | . | 1 | . | . |
| [Condition=Unfed] * [Proximity.to.Choice.1=Yes] | | 0^a^ | | . | . | | . | | . | | . | | | | . | 1 | . | . |
| C1.Meat.or.Not | | -.388 | | .3780 | -1.129 | | .353 | | 1.053 | | 1 | | | | .305 | .678 | .323 | 1.423 |
| C1.Sausage.or.Not | | -.970 | | .3799 | -1.714 | | -.225 | | 6.517 | | 1 | | | | .011 | .379 | .180 | .798 |
| C1.DryFood.or.Not | | -.157 | | .4963 | -1.129 | | .816 | | .100 | | 1 | | | | .752 | .855 | .323 | 2.261 |
| C1.Tofu.or.Not | | -.157 | | .5449 | -1.225 | | .911 | | .083 | | 1 | | | | .773 | .854 | .294 | 2.486 |
| (Scale) | | 1 | |  |  | |  | |  | |  | | | |  |  |  |  |
| Dependent Variable: C2.Chick.or.Not  Model: (Intercept), Species, Condition, Species * Condition, Proximity.to.Choice.1, Species * Proximity.to.Choice.1, Condition * Proximity.to.Choice.1, C1.Meat.or.Not, C1.Sausage.or.Not, C1.DryFood.or.Not, C1.Tofu.or.Not | | | | | | | | | | | | | | | | | | |
| a. Set to zero because this parameter is redundant. | | | | | | | | | | | | | | | | | | |

Estimated Marginal Means 1: Condition

| **Estimates** | | | | | | | | | | | | |  |  |  |  |
| --- | --- | --- | --- | --- | --- | --- | --- | --- | --- | --- | --- | --- | --- | --- | --- | --- |
| Condition | Mean | | Std. Error | | | 95% Wald Confidence Interval | | | | | | |  |  |  |  |
|  |  |  |  |  |  | Lower | | | Upper | | | |  |  |  |  |
| Fed | .85 | | .031 | | | .77 | | | .90 | | | |  |  |  |  |
| Unfed | .74 | | .046 | | | .64 | | | .82 | | | |  |  |  |  |
| Covariates appearing in the model are fixed at the following values: C1.Meat.or.Not=.35; C1.Sausage.or.Not=.22; C1.DryFood.or.Not=.06; C1.Tofu.or.Not=.11 | | | | | | | | | | | | |  |  |  |  |
| **Pairwise Comparisons** | | | | | | | | | | | | | | | |  |
| (I) Condition | | (J) Condition | | | Mean Difference (I-J) | | | Std. Error | | df | | Sequential Bonferroni Sig. | | 95% Wald Confidence Interval for Difference^b^ | |  |
|  |  |  |  |  |  |  |  |  |  |  |  |  |  | Lower | Upper |  |
| Fed | | Unfed | | | .10^a^ | | | .049 | | 1 | | .033 | | .01 | .20 |  |
| Unfed | | Fed | | | -.10^a^ | | | .049 | | 1 | | .033 | | -.20 | -.01 |  |
| Pairwise comparisons of estimated marginal means based on the original scale of dependent variable C2.Chick.or.Not | | | | | | | | | | | | | | | |  |
| a. The mean difference is significant at the .05 level. | | | | | | | | | | | | | | | |  |
| b. Confidence interval bounds are approximate. | | | | | | | | | | | | | | | |  |
| **Overall Test Results** | | | | | | | | | | |  |  |  |  |  |  |
| Wald Chi-Square | | | | df | | | Sig. | | | |  |  |  |  |  |  |
| 4.525 | | | | 1 | | | .033 | | | |  |  |  |  |  |  |
| The Wald chi-square tests the effect of Condition. This test is based on the linearly independent pairwise comparisons among the estimated marginal means. | | | | | | | | | | | | | | | | |

Estimated Marginal Means 2: Proximity to Choice 1

| **Estimates** | | | | | | | |  |  |
| --- | --- | --- | --- | --- | --- | --- | --- | --- | --- |
| Proximity.to.Choice.1 | Mean | | Std. Error | | 95% Wald Confidence Interval | | |  |  |
|  |  |  |  |  | Lower | | Upper |  |  |
| No | .75 | | .038 | | .66 | | .81 |  |  |
| Yes | .84 | | .033 | | .77 | | .90 |  |  |
| Covariates appearing in the model are fixed at the following values: C1.Meat.or.Not=.35; C1.Sausage.or.Not=.22; C1.DryFood.or.Not=.06; C1.Tofu.or.Not=.11 | | | | | | | | |  |
| **Overall Test Results** | | | | | |  |  |  |  |
| Wald Chi-Square | | df | | Sig. | |  |  |  |  |
| 6.327 | | 1 | | .012 | |  |  |  |  |
| The Wald chi-square tests the effect of Proximity.to.Choice.1. This test is based on the linearly independent pairwise comparisons among the estimated marginal means. | | | | | | | | | |

## Analysis for Meat, Choice 2

| **Model Information** | | | | | | | | | | | | |  |  |  |  |  |  |  |  |
| --- | --- | --- | --- | --- | --- | --- | --- | --- | --- | --- | --- | --- | --- | --- | --- | --- | --- | --- | --- | --- |
| Dependent Variable | | | | | C2.Meat.or.Not^a^ | | | | | | | |  |  |  |  |  |  |  |  |
| Probability Distribution | | | | | Binomial | | | | | | | |  |  |  |  |  |  |  |  |
| Link Function | | | | | Logit | | | | | | | |  |  |  |  |  |  |  |  |
| Subject Effect | 1 | | | | Animal | | | | | | | |  |  |  |  |  |  |  |  |
| Within-Subject Effect | 1 | | | | Trial | | | | | | | |  |  |  |  |  |  |  |  |
| Working Correlation Matrix Structure | | | | | Independent | | | | | | | |  |  |  |  |  |  |  |  |
| a. The procedure models 0 as the response, treating 1 as the reference category. | | | | | | | | | | | | |  |  |  |  |  |  |  |  |
| **Tests of Model Effects** | | | | | | | | | | | | | | | | | | | |  |
| Source | | | | | | Type III | | | | | | | | | | | | | |  |
|  |  |  |  |  |  | Wald Chi-Square | | | | | df | | | | | Sig. | | | |  |
| (Intercept) | | | | | | 33.683 | | | | | 1 | | | | | .000 | | | |  |
| Species | | | | | | 3.590 | | | | | 1 | | | | | .058 | | | |  |
| Condition | | | | | | .300 | | | | | 1 | | | | | .584 | | | |  |
| Proximity.to.Choice.1 | | | | | | 1.913 | | | | | 1 | | | | | .167 | | | |  |
| Species * Condition | | | | | | .005 | | | | | 1 | | | | | .944 | | | |  |
| Species * Proximity.to.Choice.1 | | | | | | 1.609 | | | | | 1 | | | | | .205 | | | |  |
| **Condition * Proximity.to.Choice.1** | | | | | | **11.934** | | | | | **1** | | | | | **.001** | | | |  |
| C1.Sausage.or.Not | | | | | | .077 | | | | | 1 | | | | | .781 | | | |  |
| C1.DryFood.or.Not | | | | | | .866 | | | | | 1 | | | | | .352 | | | |  |
| C1.Tofu.or.Not | | | | | | 3.499 | | | | | 1 | | | | | .061 | | | |  |
| C1.Chick.or.Not | | | | | | .253 | | | | | 1 | | | | | .615 | | | |  |
| Dependent Variable: C2.Meat.or.Not  Model: (Intercept), Species, Condition, Proximity.to.Choice.1, Species * Condition, Species * Proximity.to.Choice.1, Condition * Proximity.to.Choice.1, C1.Sausage.or.Not, C1.DryFood.or.Not, C1.Tofu.or.Not, C1.Chick.or.Not | | | | | | | | | | | | | | | | | | | |  |
| **Goodness of Fit^a^** | | | | | | | | | |  |  |  |  |  |  |  |  |  |  |  |
|  | | | | | | | Value | | |  |  |  |  |  |  |  |  |  |  |  |
| Quasi Likelihood under Independence Model Criterion (QIC)^b^ | | | | | | | 597.276 | | |  |  |  |  |  |  |  |  |  |  |  |
| Corrected Quasi Likelihood under Independence Model Criterion (QICC)^b^ | | | | | | | 580.427 | | |  |  |  |  |  |  |  |  |  |  |  |
| Dependent Variable: C2.Meat.or.Not  Model: (Intercept), Species, Condition, Proximity.to.Choice.1, Species * Condition, Species * Proximity.to.Choice.1, Condition * Proximity.to.Choice.1, C1.Sausage.or.Not, C1.DryFood.or.Not, C1.Tofu.or.Not, C1.Chick.or.Not^a^ | | | | | | | | | | | | | | | | | | |  |  |
| a. Information criteria are in smaller-is-better form. | | | | | | | | | | | | | | | | | | |  |  |
| b. Computed using the full log quasi-likelihood function. | | | | | | | | | | | | | | | | | | |  |  |
| **Parameter Estimates** | | | | | | | | | | | | | | | | | | | | |
| Parameter | | B | Std. Error | 95% Wald Confidence Interval | | | | | Hypothesis Test | | | | | | Exp(B) | | 95% Wald Confidence Interval for Exp(B) | | | |
|  |  |  |  | Lower | | | | Upper | Wald Chi-Square | | | df | | Sig. |  |  | Lower | Upper | | |
| (Intercept) | | 1.709 | .4281 | .870 | | | | 2.548 | 15.945 | | | 1 | | .000 | 5.525 | | 2.388 | 12.786 | | |
| [Species=Dog] | | .916 | .6380 | -.334 | | | | 2.167 | 2.063 | | | 1 | | .151 | 2.500 | | .716 | 8.732 | | |
| [Species=Wolf] | | 0^a^ | . | . | | | | . | . | | | . | | . | 1 | | . | . | | |
| [Condition=Fed] | | -1.068 | .5440 | -2.134 | | | | -.002 | 3.855 | | | 1 | | .050 | .344 | | .118 | .998 | | |
| [Condition=Unfed] | | 0^a^ | . | . | | | | . | . | | | . | | . | 1 | | . | . | | |
| [Proximity.to.Choice.1=No] | | -.946 | .5095 | -1.945 | | | | .052 | 3.448 | | | 1 | | .063 | .388 | | .143 | 1.054 | | |
| [Proximity.to.Choice.1=Yes] | | 0^a^ | . | . | | | | . | . | | | . | | . | 1 | | . | . | | |
| [Species=Dog] * [Condition=Fed] | | -.052 | .7312 | -1.485 | | | | 1.381 | .005 | | | 1 | | .944 | .950 | | .227 | 3.981 | | |
| [Species=Dog] * [Condition=Unfed] | | 0^a^ | . | . | | | | . | . | | | . | | . | 1 | | . | . | | |
| [Species=Wolf] * [Condition=Fed] | | 0^a^ | . | . | | | | . | . | | | . | | . | 1 | | . | . | | |
| [Species=Wolf] * [Condition=Unfed] | | 0^a^ | . | . | | | | . | . | | | . | | . | 1 | | . | . | | |
| [Species=Dog] * [Proximity.to.Choice.1=No] | | -.800 | .6304 | -2.035 | | | | .436 | 1.609 | | | 1 | | .205 | .449 | | .131 | 1.546 | | |
| [Species=Dog] * [Proximity.to.Choice.1=Yes] | | 0^a^ | . | . | | | | . | . | | | . | | . | 1 | | . | . | | |
| [Species=Wolf] * [Proximity.to.Choice.1=No] | | 0^a^ | . | . | | | | . | . | | | . | | . | 1 | | . | . | | |
| [Species=Wolf] * [Proximity.to.Choice.1=Yes] | | 0^a^ | . | . | | | | . | . | | | . | | . | 1 | | . | . | | |
| [Condition=Fed] * [Proximity.to.Choice.1=No] | | 1.808 | .5233 | .782 | | | | 2.834 | 11.934 | | | 1 | | .001 | 6.097 | | 2.186 | 17.005 | | |
| [Condition=Fed] * [Proximity.to.Choice.1=Yes] | | 0^a^ | . | . | | | | . | . | | | . | | . | 1 | | . | . | | |
| [Condition=Unfed] * [Proximity.to.Choice.1=No] | | 0^a^ | . | . | | | | . | . | | | . | | . | 1 | | . | . | | |
| [Condition=Unfed] * [Proximity.to.Choice.1=Yes] | | 0^a^ | . | . | | | | . | . | | | . | | . | 1 | | . | . | | |
| C1.Sausage.or.Not | | -.104 | .3764 | -.842 | | | | .633 | .077 | | | 1 | | .781 | .901 | | .431 | 1.884 | | |
| C1.DryFood.or.Not | | .421 | .4520 | -.465 | | | | 1.307 | .866 | | | 1 | | .352 | 1.523 | | .628 | 3.693 | | |
| C1.Tofu.or.Not | | -.911 | .4869 | -1.865 | | | | .043 | 3.499 | | | 1 | | .061 | .402 | | .155 | 1.044 | | |
| C1.Chick.or.Not | | .168 | .3329 | -.485 | | | | .820 | .253 | | | 1 | | .615 | 1.182 | | .616 | 2.270 | | |
| (Scale) | | 1 |  |  | | | |  |  | | |  | |  |  | |  |  | | |
| Dependent Variable: C2.Meat.or.Not  Model: (Intercept), Species, Condition, Proximity.to.Choice.1, Species * Condition, Species * Proximity.to.Choice.1, Condition * Proximity.to.Choice.1, C1.Sausage.or.Not, C1.DryFood.or.Not, C1.Tofu.or.Not, C1.Chick.or.Not | | | | | | | | | | | | | | | | | | | | |
| a. Set to zero because this parameter is redundant. | | | | | | | | | | | | | | | | | | | | |

Estimated Marginal Means: Condition * Proximity to Choice 1

| **Estimates** | | | | | | | | | | | | | | |  |  |  |  |
| --- | --- | --- | --- | --- | --- | --- | --- | --- | --- | --- | --- | --- | --- | --- | --- | --- | --- | --- |
| Condition | Proximity.to.Choice.1 | | | | Mean | | | Std. Error | | 95% Wald Confidence Interval | | | | |  |  |  |  |
|  |  |  |  |  |  |  |  |  |  | Lower | | Upper | | |  |  |  |  |
| Fed | No | | | | .81 | | | .041 | | .72 | | .88 | | |  |  |  |  |
|  | Yes | | | | .73 | | | .057 | | .61 | | .83 | | |  |  |  |  |
| Unfed | No | | | | .68 | | | .053 | | .57 | | .78 | | |  |  |  |  |
|  | Yes | | | | .89 | | | .037 | | .79 | | .95 | | |  |  |  |  |
| Covariates appearing in the model are fixed at the following values: C1.Sausage.or.Not=.22; C1.DryFood.or.Not=.06; C1.Tofu.or.Not=.11; C1.Chick.or.Not=.24 | | | | | | | | | | | | | | |  |  |  |  |
| **Pairwise Comparisons** | | | | | | | | | | | | | | | | | | |
| (I) Condition * Proximity.to.Choice.1 | | | | (J) Condition * Proximity.to.Choice.1 | | | | | Mean Difference (I-J) | | Std. Error | | df | Sequential Bonferroni Sig. | | 95% Wald Confidence Interval for Difference^a^ | | |
|  |  |  |  |  |  |  |  |  |  |  |  |  |  |  |  | Lower | Upper | |
| [Condition=Fed] * [Proximity.to.Choice.1=No] | | | | [Condition=Fed] * [Proximity.to.Choice.1=Yes] | | | | | .08 | | .063 | | 1 | .489 | | -.07 | .23 | |
|  |  |  |  | [Condition=Unfed] * [Proximity.to.Choice.1=No] | | | | | .13 | | .080 | | 1 | .396 | | -.07 | .33 | |
|  |  |  |  | [Condition=Unfed] * [Proximity.to.Choice.1=Yes] | | | | | -.08 | | .056 | | 1 | .489 | | -.21 | .06 | |
| [Condition=Fed] * [Proximity.to.Choice.1=Yes] | | | | [Condition=Fed] * [Proximity.to.Choice.1=No] | | | | | -.08 | | .063 | | 1 | .489 | | -.23 | .07 | |
|  |  |  |  | [Condition=Unfed] * [Proximity.to.Choice.1=No] | | | | | .05 | | .096 | | 1 | .588 | | -.14 | .24 | |
|  |  |  |  | [Condition=Unfed] * [Proximity.to.Choice.1=Yes] | | | | | **-.16^b^** | | **.061** | | **1** | **.048** | | -.31 | .00 | |
| [Condition=Unfed] * [Proximity.to.Choice.1=No] | | | | [Condition=Fed] * [Proximity.to.Choice.1=No] | | | | | -.13 | | .080 | | 1 | .396 | | -.33 | .07 | |
|  |  |  |  | [Condition=Fed] * [Proximity.to.Choice.1=Yes] | | | | | -.05 | | .096 | | 1 | .588 | | -.24 | .14 | |
|  |  |  |  | [Condition=Unfed] * [Proximity.to.Choice.1=Yes] | | | | | **-.21^b^** | | **.065** | | **1** | **.008** | | -.38 | -.04 | |
| [Condition=Unfed] * [Proximity.to.Choice.1=Yes] | | | | [Condition=Fed] * [Proximity.to.Choice.1=No] | | | | | .08 | | .056 | | 1 | .489 | | -.06 | .21 | |
|  |  |  |  | [Condition=Fed] * [Proximity.to.Choice.1=Yes] | | | | | **.16^b^** | | **.061** | | **1** | **.048** | | .00 | .31 | |
|  |  |  |  | [Condition=Unfed] * [Proximity.to.Choice.1=No] | | | | | **.21^b^** | | **.065** | | **1** | **.008** | | .04 | .38 | |
| Pairwise comparisons of estimated marginal means based on the original scale of dependent variable C2.Meat.or.Not | | | | | | | | | | | | | | | | | | |
| a. Confidence interval bounds are approximate. | | | | | | | | | | | | | | | | | | |
| b. The mean difference is significant at the .05 level. | | | | | | | | | | | | | | | | | | |
| **Overall Test Results** | | | | | | |  |  |  |  |  |  |  |  |  |  |  |  |
| Wald Chi-Square | | df | Sig. | | |  |  |  |  |  |  |  |  |  |  |  |  |  |
| 20.173 | | 3 | .000 | | |  |  |  |  |  |  |  |  |  |  |  |  |  |
| The Wald chi-square tests the effect of Condition*Proximity.to.Choice.1. This test is based on the linearly independent pairwise comparisons among the estimated marginal means. | | | | | | | | | | | | | | | | | |  |

## Analysis for Sausage, Choice 2

| **Model Information** | | | | |  |  |  |  |
| --- | --- | --- | --- | --- | --- | --- | --- | --- |
| Dependent Variable | | C2.Sausage.or.Not^a^ | | |  |  |  |  |
| Probability Distribution | | Binomial | | |  |  |  |  |
| Link Function | | Logit | | |  |  |  |  |
| Subject Effect | 1 | Animal | | |  |  |  |  |
| Within-Subject Effect | 1 | Trial | | |  |  |  |  |
| Working Correlation Matrix Structure | | Independent | | |  |  |  |  |
| a. The procedure models 0 as the response, treating 1 as the reference category. | | | | | |  |  |  |
| **Tests of Model Effects** | | | | | | |  |  |
| Source | Type III | | | | | |  |  |
|  | Wald Chi-Square | | df | | Sig. | |  |  |
| (Intercept) | 57.144 | | 1 | | .000 | |  |  |
| Species | .559 | | 1 | | .455 | |  |  |
| Condition | 1.322 | | 1 | | .250 | |  |  |
| **Proximity.to.Choice.1** | **10.714** | | **1** | | **.001** | |  |  |
| Species * Condition | .291 | | 1 | | .589 | |  |  |
| Species * Proximity.to.Choice.1 | 1.999 | | 1 | | .157 | |  |  |
| Condition * Proximity.to.Choice.1 | 1.393 | | 1 | | .238 | |  |  |
| **C1.DryFood.or.Not** | **11.024** | | **1** | | **.001** | |  |  |
| C1.Tofu.or.Not | 2.348 | | 1 | | .125 | |  |  |
| **C1.Chick.or.Not** | **9.731** | | **1** | | **.002** | |  |  |
| **C1.Meat.or.Not** | **7.699** | | **1** | | **.006** | |  |  |
| Dependent Variable: C2.Sausage.or.Not  Model: (Intercept), Species, Condition, Proximity.to.Choice.1, Species * Condition, Species * Proximity.to.Choice.1, Condition * Proximity.to.Choice.1, C1.DryFood.or.Not, C1.Tofu.or.Not, C1.Chick.or.Not, C1.Meat.or.Not | | | | | | | | |
| **Goodness of Fit^a^** | | | | | |  |  |  |
|  | | | | Value | |  |  |  |
| Quasi Likelihood under Independence Model Criterion (QIC)^b^ | | | | 591.670 | |  |  |  |
| Corrected Quasi Likelihood under Independence Model Criterion (QICC)^b^ | | | | 579.601 | |  |  |  |
| Dependent Variable: C2.Sausage.or.Not  Model: (Intercept), Species, Condition, Proximity.to.Choice.1, Species * Condition, Species * Proximity.to.Choice.1, Condition * Proximity.to.Choice.1, C1.DryFood.or.Not, C1.Tofu.or.Not, C1.Chick.or.Not, C1.Meat.or.Not^a^ | | | | | | | |  |
| a. Information criteria are in smaller-is-better form. | | | | | | | |  |
| b. Computed using the full log quasi-likelihood function. | | | | | | | |  |

| **Parameter Estimates** | | | | | | | | | | |
| --- | --- | --- | --- | --- | --- | --- | --- | --- | --- | --- |
| Parameter | B | Std. Error | 95% Wald Confidence Interval | | Hypothesis Test | | | Exp(B) | 95% Wald Confidence Interval for Exp(B) | |
|  |  |  | Lower | Upper | Wald Chi-Square | df | Sig. |  | Lower | Upper |
| (Intercept) | 2.485 | .4791 | 1.546 | 3.424 | 26.904 | 1 | .000 | 12.000 | 4.692 | 30.688 |
| [Species=Dog] | -.074 | .5750 | -1.201 | 1.053 | .016 | 1 | .898 | .929 | .301 | 2.867 |
| [Species=Wolf] | 0^a^ | . | . | . | . | . | . | 1 | . | . |
| [Condition=Fed] | .208 | .4038 | -.583 | 1.000 | .266 | 1 | .606 | 1.231 | .558 | 2.717 |
| [Condition=Unfed] | 0^a^ | . | . | . | . | . | . | 1 | . | . |
| [Proximity.to.Choice.1=No] | -1.144 | .5260 | -2.175 | -.113 | 4.729 | 1 | .030 | .319 | .114 | .893 |
| [Proximity.to.Choice.1=Yes] | 0^a^ | . | . | . | . | . | . | 1 | . | . |
| [Species=Dog] * [Condition=Fed] | -.250 | .4627 | -1.157 | .657 | .291 | 1 | .589 | .779 | .315 | 1.929 |
| [Species=Dog] * [Condition=Unfed] | 0^a^ | . | . | . | . | . | . | 1 | . | . |
| [Species=Wolf] * [Condition=Fed] | 0^a^ | . | . | . | . | . | . | 1 | . | . |
| [Species=Wolf] * [Condition=Unfed] | 0^a^ | . | . | . | . | . | . | 1 | . | . |
| [Species=Dog] * [Proximity.to.Choice.1=No] | .886 | .6266 | -.342 | 2.114 | 1.999 | 1 | .157 | 2.425 | .710 | 8.281 |
| [Species=Dog] * [Proximity.to.Choice.1=Yes] | 0^a^ | . | . | . | . | . | . | 1 | . | . |
| [Species=Wolf] * [Proximity.to.Choice.1=No] | 0^a^ | . | . | . | . | . | . | 1 | . | . |
| [Species=Wolf] * [Proximity.to.Choice.1=Yes] | 0^a^ | . | . | . | . | . | . | 1 | . | . |
| [Condition=Fed] * [Proximity.to.Choice.1=No] | -.668 | .5659 | -1.777 | .441 | 1.393 | 1 | .238 | .513 | .169 | 1.555 |
| [Condition=Fed] * [Proximity.to.Choice.1=Yes] | 0^a^ | . | . | . | . | . | . | 1 | . | . |
| [Condition=Unfed] * [Proximity.to.Choice.1=No] | 0^a^ | . | . | . | . | . | . | 1 | . | . |
| [Condition=Unfed] * [Proximity.to.Choice.1=Yes] | 0^a^ | . | . | . | . | . | . | 1 | . | . |
| C1.DryFood.or.Not | -1.404 | .4228 | -2.232 | -.575 | 11.024 | 1 | .001 | .246 | .107 | .563 |
| C1.Tofu.or.Not | -.642 | .4188 | -1.463 | .179 | 2.348 | 1 | .125 | .526 | .232 | 1.196 |
| C1.Chick.or.Not | -1.021 | .3271 | -1.662 | -.379 | 9.731 | 1 | .002 | .360 | .190 | .684 |
| C1.Meat.or.Not | -.785 | .2830 | -1.340 | -.231 | 7.699 | 1 | .006 | .456 | .262 | .794 |
| (Scale) | 1 |  |  |  |  |  |  |  |  |  |
| Dependent Variable: C2.Sausage.or.Not  Model: (Intercept), Species, Condition, Proximity.to.Choice.1, Species * Condition, Species * Proximity.to.Choice.1, Condition * Proximity.to.Choice.1, C1.DryFood.or.Not, C1.Tofu.or.Not, C1.Chick.or.Not, C1.Meat.or.Not | | | | | | | | | | |
| a. Set to zero because this parameter is redundant. | | | | | | | | | | |

## Analysis for Dry Food, Choice 2

| **Model Information** | | | | | |  |  |  |  |  |
| --- | --- | --- | --- | --- | --- | --- | --- | --- | --- | --- |
| Dependent Variable | | C2.DryFood.or.Not^a^ | | | |  |  |  |  |  |
| Probability Distribution | | Binomial | | | |  |  |  |  |  |
| Link Function | | Logit | | | |  |  |  |  |  |
| Subject Effect | 1 | Animal | | | |  |  |  |  |  |
| Within-Subject Effect | 1 | Trial | | | |  |  |  |  |  |
| Working Correlation Matrix Structure | | Independent | | | |  |  |  |  |  |
| a. The procedure models 0 as the response, treating 1 as the reference category. | | | | | | | | |  |  |
| **Tests of Model Effects** | | | | | | | | |  |  |
| Source | Type III | | | | | | | |  |  |
|  | Wald Chi-Square | | df | | Sig. | | | |  |  |
| (Intercept) | 22.408 | | 1 | | .000 | | | |  |  |
| Species | .971 | | 1 | | .324 | | | |  |  |
| Condition | .017 | | 1 | | .897 | | | |  |  |
| Proximity.to.Choice.1 | 3.858 | | 1 | | .050 | | | |  |  |
| Species * Condition | .056 | | 1 | | .813 | | | |  |  |
| Species * Proximity.to.Choice.1 | .092 | | 1 | | .761 | | | |  |  |
| Condition * Proximity.to.Choice.1 | .402 | | 1 | | .526 | | | |  |  |
| C1.Tofu.or.Not | .042 | | 1 | | .837 | | | |  |  |
| C1.Chick.or.Not | 2.290 | | 1 | | .130 | | | |  |  |
| C1.Meat.or.Not | 2.109 | | 1 | | .146 | | | |  |  |
| C1.Sausage.or.Not | .419 | | 1 | | .517 | | | |  |  |
| Dependent Variable: C2.DryFood.or.Not  Model: (Intercept), Species, Condition, Proximity.to.Choice.1, Species * Condition, Species * Proximity.to.Choice.1, Condition * Proximity.to.Choice.1, C1.Tofu.or.Not, C1.Chick.or.Not, C1.Meat.or.Not, C1.Sausage.or.Not | | | | | | | | | |  |
| **Goodness of Fit^a^** | | | | | | |  |  |  |  |
|  | | | | Value | | | |  |  |  |
| Quasi Likelihood under Independence Model Criterion (QIC)^b^ | | | | 447.317 | | | |  |  |  |
| Corrected Quasi Likelihood under Independence Model Criterion (QICC)^b^ | | | | 436.037 | | | |  |  |  |
| Dependent Variable: C2.DryFood.or.Not  Model: (Intercept), Species, Condition, Proximity.to.Choice.1, Species * Condition, Species * Proximity.to.Choice.1, Condition * Proximity.to.Choice.1, C1.Tofu.or.Not, C1.Chick.or.Not, C1.Meat.or.Not, C1.Sausage.or.Not^a^ | | | | | | | | | | |
| a. Information criteria are in smaller-is-better form. | | | | | | | | | | |
| b. Computed using the full log quasi-likelihood function. | | | | | | | | | | |

| **Parameter Estimates** | | | | | | | | | | | |
| --- | --- | --- | --- | --- | --- | --- | --- | --- | --- | --- | --- |
| Parameter | B | | Std. Error | 95% Wald Confidence Interval | | Hypothesis Test | | | Exp(B) | 95% Wald Confidence Interval for Exp(B) | |
|  |  |  |  | Lower | Upper | Wald Chi-Square | df | Sig. |  | Lower | Upper |
| (Intercept) | 2.418 | | .6222 | 1.198 | 3.637 | 15.100 | 1 | .000 | 11.221 | 3.314 | 37.987 |
| [Species=Dog] | -.487 | | .5638 | -1.592 | .619 | .745 | 1 | .388 | .615 | .204 | 1.856 |
| [Species=Wolf] | 0^a^ | | . | . | . | . | . | . | 1 | . | . |
| [Condition=Fed] | .228 | | .4404 | -.635 | 1.091 | .269 | 1 | .604 | 1.256 | .530 | 2.978 |
| [Condition=Unfed] | 0^a^ | | . | . | . | . | . | . | 1 | . | . |
| [Proximity.to.Choice.1=No] | .881 | | .7941 | -.675 | 2.438 | 1.232 | 1 | .267 | 2.414 | .509 | 11.448 |
| [Proximity.to.Choice.1=Yes] | 0^a^ | | . | . | . | . | . | . | 1 | . | . |
| [Species=Dog] * [Condition=Fed] | -.141 | | .5960 | -1.309 | 1.027 | .056 | 1 | .813 | .868 | .270 | 2.792 |
| [Species=Dog] * [Condition=Unfed] | 0^a^ | | . | . | . | . | . | . | 1 | . | . |
| [Species=Wolf] * [Condition=Fed] | 0^a^ | | . | . | . | . | . | . | 1 | . | . |
| [Species=Wolf] * [Condition=Unfed] | 0^a^ | | . | . | . | . | . | . | 1 | . | . |
| [Species=Dog] * [Proximity.to.Choice.1=No] | .252 | | .8311 | -1.376 | 1.881 | .092 | 1 | .761 | 1.287 | .252 | 6.562 |
| [Species=Dog] * [Proximity.to.Choice.1=Yes] | 0^a^ | | . | . | . | . | . | . | 1 | . | . |
| [Species=Wolf] * [Proximity.to.Choice.1=No] | 0^a^ | | . | . | . | . | . | . | 1 | . | . |
| [Species=Wolf] * [Proximity.to.Choice.1=Yes] | 0^a^ | | . | . | . | . | . | . | 1 | . | . |
| [Condition=Fed] * [Proximity.to.Choice.1=No] | -.389 | | .6134 | -1.591 | .813 | .402 | 1 | .526 | .678 | .204 | 2.255 |
| [Condition=Fed] * [Proximity.to.Choice.1=Yes] | 0^a^ | | . | . | . | . | . | . | 1 | . | . |
| [Condition=Unfed] * [Proximity.to.Choice.1=No] | 0^a^ | | . | . | . | . | . | . | 1 | . | . |
| [Condition=Unfed] * [Proximity.to.Choice.1=Yes] | 0^a^ | | . | . | . | . | . | . | 1 | . | . |
| C1.Tofu.or.Not | -.133 | | .6451 | -1.397 | 1.131 | .042 | 1 | .837 | .876 | .247 | 3.100 |
| C1.Chick.or.Not | -.776 | | .5129 | -1.782 | .229 | 2.290 | 1 | .130 | .460 | .168 | 1.257 |
| C1.Meat.or.Not | -.836 | | .5755 | -1.964 | .292 | 2.109 | 1 | .146 | .434 | .140 | 1.339 |
| C1.Sausage.or.Not | -.389 | .6011 | | -1.567 | .789 | .419 | 1 | .517 | .678 | .209 | 2.201 |
| (Scale) | 1 |  | |  |  |  |  |  |  |  |  |
| Dependent Variable: C2.DryFood.or.Not  Model: (Intercept), Species, Condition, Proximity.to.Choice.1, Species * Condition, Species * Proximity.to.Choice.1, Condition * Proximity.to.Choice.1, C1.Tofu.or.Not, C1.Chick.or.Not, C1.Meat.or.Not, C1.Sausage.or.Not | | | | | | | | | | | |
| a. Set to zero because this parameter is redundant. | | | | | | | | | | | |

## Analysis for Tofu, Choice 2

| **Model Information** | | | | | | | | | | | | |  |  |  |  |  |  |  |  |  |
| --- | --- | --- | --- | --- | --- | --- | --- | --- | --- | --- | --- | --- | --- | --- | --- | --- | --- | --- | --- | --- | --- |
| Dependent Variable | | | | | C2.Tofu.or.Not^a^ | | | | | | | |  |  |  |  |  |  |  |  |  |
| Probability Distribution | | | | | Binomial | | | | | | | |  |  |  |  |  |  |  |  |  |
| Link Function | | | | | Logit | | | | | | | |  |  |  |  |  |  |  |  |  |
| Subject Effect | | | 1 | | Animal | | | | | | | |  |  |  |  |  |  |  |  |  |
| Within-Subject Effect | | | 1 | | Trial | | | | | | | |  |  |  |  |  |  |  |  |  |
| Working Correlation Matrix Structure | | | | | Independent | | | | | | | |  |  |  |  |  |  |  |  |  |
| a. The procedure models 0 as the response, treating 1 as the reference category. | | | | | | | | | | | | | | | | |  |  |  |  |  |
| **Tests of Model Effects** | | | | | | | | | | | | |  |  |  |  |  |  |  |  |  |
| Source | | Type III | | | | | | | | | | |  |  |  |  |  |  |  |  |  |
|  |  | Wald Chi-Square | | | | | | df | | Sig. | | |  |  |  |  |  |  |  |  |  |
| (Intercept) | | 23.398 | | | | | | 1 | | .000 | | |  |  |  |  |  |  |  |  |  |
| **Species** | | **6.041** | | | | | | **1** | | **.014** | | |  |  |  |  |  |  |  |  |  |
| **Condition** | | **5.318** | | | | | | **1** | | **.021** | | |  |  |  |  |  |  |  |  |  |
| **Proximity.to.Choice.1** | | **19.681** | | | | | | **1** | | **.000** | | |  |  |  |  |  |  |  |  |  |
| **Species * Condition** | | **4.523** | | | | | | **1** | | **.033** | | |  |  |  |  |  |  |  |  |  |
| Species * Proximity.to.Choice.1 | | 1.243 | | | | | | 1 | | .265 | | |  |  |  |  |  |  |  |  |  |
| Condition * Proximity.to.Choice.1 | | 2.792 | | | | | | 1 | | .095 | | |  |  |  |  |  |  |  |  |  |
| C1.Chick.or.Not | | .540 | | | | | | 1 | | .463 | | |  |  |  |  |  |  |  |  |  |
| C1.Meat.or.Not | | .258 | | | | | | 1 | | .611 | | |  |  |  |  |  |  |  |  |  |
| C1.Sausage.or.Not | | .108 | | | | | | 1 | | .742 | | |  |  |  |  |  |  |  |  |  |
| C1.DryFood.or.Not | | .584 | | | | | | 1 | | .445 | | |  |  |  |  |  |  |  |  |  |
| Dependent Variable: C2.Tofu.or.Not  Model: (Intercept), Species, Condition, Proximity.to.Choice.1, Species * Condition, Species * Proximity.to.Choice.1, Condition * Proximity.to.Choice.1, C1.Chick.or.Not, C1.Meat.or.Not, C1.Sausage.or.Not, C1.DryFood.or.Not | | | | | | | | | | | | | | | | | | | |  |  |
| **Goodness of Fit^a^** | | | | | | | | | | | | |  |  |  |  |  |  |  |  |  |
|  | | | | | | | Value | | | | |  |  |  |  |  |  |  |  |  |  |
| Quasi Likelihood under Independence Model Criterion (QIC)^b^ | | | | | | | 434.706 | | | | |  |  |  |  |  |  |  |  |  |  |
| Corrected Quasi Likelihood under Independence Model Criterion (QICC)^b^ | | | | | | | 418.394 | | | | |  |  |  |  |  |  |  |  |  |  |
| Dependent Variable: C2.Tofu.or.Not  Model: (Intercept), Species, Condition, Proximity.to.Choice.1, Species * Condition, Species * Proximity.to.Choice.1, Condition * Proximity.to.Choice.1, C1.Chick.or.Not, C1.Meat.or.Not, C1.Sausage.or.Not, C1.DryFood.or.Not^a^ | | | | | | | | | | | | | | | | | | | | |  |
| a. Information criteria are in smaller-is-better form. | | | | | | | | | | | | | | | | | | | | |  |
| b. Computed using the full log quasi-likelihood function. | | | | | | | | | | | | | | | | | | | | |  |
| **Parameter Estimates** | | | | | | | | | | | | | | | | | | | | | |
| Parameter | B | | | Std. Error | | 95% Wald Confidence Interval | | | | | Hypothesis Test | | | | | Exp(B) | | 95% Wald Confidence Interval for Exp(B) | | | |
|  |  |  |  |  |  | Lower | | | Upper | | Wald Chi-Square | | | df | Sig. |  |  | Lower | Upper | | |
| (Intercept) | 1.868 | | | .4561 | | .974 | | | 2.762 | | 16.779 | | | 1 | .000 | 6.476 | | 2.649 | 15.831 | | |
| [Species=Dog] | -1.500 | | | .5674 | | -2.612 | | | -.388 | | 6.990 | | | 1 | .008 | .223 | | .073 | .678 | | |
| [Species=Wolf] | 0^a^ | | | . | | . | | | . | | . | | | . | . | 1 | | . | . | | |
| [Condition=Fed] | -1.305 | | | .6396 | | -2.558 | | | -.051 | | 4.162 | | | 1 | .041 | .271 | | .077 | .950 | | |
| [Condition=Unfed] | 0^a^ | | | . | | . | | | . | | . | | | . | . | 1 | | . | . | | |
| [Proximity.to.Choice.1=No] | 4.314 | | | 1.3367 | | 1.694 | | | 6.934 | | 10.417 | | | 1 | .001 | 74.747 | | 5.443 | 1026.537 | | |
| [Proximity.to.Choice.1=Yes] | 0^a^ | | | . | | . | | | . | | . | | | . | . | 1 | | . | . | | |
| [Species=Dog] * [Condition=Fed] | 1.826 | | | .8586 | | .143 | | | 3.509 | | 4.523 | | | 1 | .033 | 6.209 | | 1.154 | 33.413 | | |
| [Species=Dog] * [Condition=Unfed] | 0^a^ | | | . | | . | | | . | | . | | | . | . | 1 | | . | . | | |
| [Species=Wolf] * [Condition=Fed] | 0^a^ | | | . | | . | | | . | | . | | | . | . | 1 | | . | . | | |
| [Species=Wolf] * [Condition=Unfed] | 0^a^ | | | . | | . | | | . | | . | | | . | . | 1 | | . | . | | |
| [Species=Dog] * [Proximity.to.Choice.1=No] | -1.290 | | | 1.1572 | | -3.558 | | | .978 | | 1.243 | | | 1 | .265 | .275 | | .028 | 2.659 | | |
| [Species=Dog] * [Proximity.to.Choice.1=Yes] | 0^a^ | | | . | | . | | | . | | . | | | . | . | 1 | | . | . | | |
| [Species=Wolf] * [Proximity.to.Choice.1=No] | 0^a^ | | | . | | . | | | . | | . | | | . | . | 1 | | . | . | | |
| [Species=Wolf] * [Proximity.to.Choice.1=Yes] | 0^a^ | | | . | | . | | | . | | . | | | . | . | 1 | | . | . | | |
| [Condition=Fed] * [Proximity.to.Choice.1=No] | -1.578 | | | .9445 | | -3.430 | | | .273 | | 2.792 | | | 1 | .095 | .206 | | .032 | 1.314 | | |
| [Condition=Fed] * [Proximity.to.Choice.1=Yes] | 0^a^ | | | . | | . | | | . | | . | | | . | . | 1 | | . | . | | |
| [Condition=Unfed] * [Proximity.to.Choice.1=No] | 0^a^ | | | . | | . | | | . | | . | | | . | . | 1 | | . | . | | |
| [Condition=Unfed] * [Proximity.to.Choice.1=Yes] | 0^a^ | | | . | | . | | | . | | . | | | . | . | 1 | | . | . | | |
| C1.Chick.or.Not | .373 | | | .5083 | | -.623 | | | 1.370 | | .540 | | | 1 | .463 | 1.453 | | .536 | 3.934 | | |
| C1.Meat.or.Not | .295 | | | .5808 | | -.843 | | | 1.434 | | .258 | | | 1 | .611 | 1.343 | | .430 | 4.194 | | |
| C1.Sausage.or.Not | .159 | | | .4835 | | -.789 | | | 1.106 | | .108 | | | 1 | .742 | 1.172 | | .454 | 3.024 | | |
| C1.DryFood.or.Not | .657 | | | .8593 | | -1.027 | | | 2.341 | | .584 | | | 1 | .445 | 1.929 | | .358 | 10.391 | | |
| (Scale) | 1 | | |  | |  | | |  | |  | | |  |  |  | |  |  | | |
| Dependent Variable: C2.Tofu.or.Not  Model: (Intercept), Species, Condition, Proximity.to.Choice.1, Species * Condition, Species * Proximity.to.Choice.1, Condition * Proximity.to.Choice.1, C1.Chick.or.Not, C1.Meat.or.Not, C1.Sausage.or.Not, C1.DryFood.or.Not | | | | | | | | | | | | | | | | | | | | | |
| a. Set to zero because this parameter is redundant. | | | | | | | | | | | | | | | | | | | | | |

Estimated Marginal Means: Species* Condition

| **Estimates** | | | | | | | | | | | |  |  |  |  |  |  |  |
| --- | --- | --- | --- | --- | --- | --- | --- | --- | --- | --- | --- | --- | --- | --- | --- | --- | --- | --- |
| Species | Condition | | Mean | | | Std. Error | | 95% Wald Confidence Interval | | | |  |  |  |  |  |  |  |
|  |  |  |  |  |  |  |  | Lower | Upper | | |  |  |  |  |  |  |  |
| Dog | Fed | | .87 | | | .037 | | .78 | .93 | | |  |  |  |  |  |  |  |
|  | Unfed | | .90 | | | .035 | | .80 | .95 | | |  |  |  |  |  |  |  |
| Wolf | Fed | | .90 | | | .051 | | .75 | .97 | | |  |  |  |  |  |  |  |
|  | Unfed | | .99 | | | .010 | | .95 | 1.00 | | |  |  |  |  |  |  |  |
| Covariates appearing in the model are fixed at the following values: C1.Chick.or.Not=.24; C1.Meat.or.Not=.35; C1.Sausage.or.Not=.22; C1.DryFood.or.Not=.06 | | | | | | | | | | | | | | | | |  |  |
| **Pairwise Comparisons** | | | | | | | | | | | | | | | | | |  |
| (I) Species*Condition | | | | (J) Species*Condition | | | | | | Mean Difference (I-J) | Std. Error | | df | Sequential Bonferroni Sig. | 95% Wald Confidence Interval for Difference^a^ | | |  |
|  |  |  |  |  |  |  |  |  |  |  |  |  |  |  | Lower | Upper | |  |
| [Species=Dog] * [Condition=Fed] | | | | [Species=Dog] * [Condition=Unfed] | | | | | | -.03 | .056 | | 1 | 1.000 | -.15 | .09 | |  |
|  |  |  |  | [Species=Wolf] * [Condition=Fed] | | | | | | -.03 | .057 | | 1 | 1.000 | -.17 | .10 | |  |
|  |  |  |  | [Species=Wolf] * [Condition=Unfed] | | | | | | -.12^b^ | .037 | | 1 | .009 | -.22 | -.02 | |  |
| [Species=Dog] * [Condition=Unfed] | | | | [Species=Dog] * [Condition=Fed] | | | | | | .03 | .056 | | 1 | 1.000 | -.09 | .15 | |  |
|  |  |  |  | [Species=Wolf] * [Condition=Fed] | | | | | | .00 | .061 | | 1 | 1.000 | -.13 | .12 | |  |
|  |  |  |  | [Species=Wolf] * [Condition=Unfed] | | | | | | **-.09^b^** | **.034** | | **1** | **.035** | -.18 | .00 | |  |
| [Species=Wolf] * [Condition=Fed] | | | | [Species=Dog] * [Condition=Fed] | | | | | | .03 | .057 | | 1 | 1.000 | -.10 | .17 | |  |
|  |  |  |  | [Species=Dog] * [Condition=Unfed] | | | | | | .00 | .061 | | 1 | 1.000 | -.12 | .13 | |  |
|  |  |  |  | [Species=Wolf] * [Condition=Unfed] | | | | | | -.09 | .049 | | 1 | .316 | -.21 | .04 | |  |
| [Species=Wolf] * [Condition=Unfed] | | | | [Species=Dog] * [Condition=Fed] | | | | | | .12^b^ | .037 | | 1 | .009 | .02 | .22 | |  |
|  |  |  |  | [Species=Dog] * [Condition=Unfed] | | | | | | **.09^b^** | **.034** | | **1** | **.035** | .00 | .18 | |  |
|  |  |  |  | [Species=Wolf] * [Condition=Fed] | | | | | | .09 | .049 | | 1 | .316 | -.04 | .21 | |  |
| Pairwise comparisons of estimated marginal means based on the original scale of dependent variable C2.Tofu.or.Not | | | | | | | | | | | | | | | | | |  |
| a. Confidence interval bounds are approximate. | | | | | | | | | | | | | | | | | |  |
| b. The mean difference is significant at the .05 level. | | | | | | | | | | | | | | | | | |  |
| **Overall Test Results** | | | | | | |  |  |  |  |  |  |  |  |  |  |  |  |
| Wald Chi-Square | | df | | | Sig. | |  |  |  |  |  |  |  |  |  |  |  |  |
| 24.174 | | 3 | | | .000 | |  |  |  |  |  |  |  |  |  |  |  |  |
| The Wald chi-square tests the effect of Species*Condition. This test is based on the linearly independent pairwise comparisons among the estimated marginal means. | | | | | | | | | | | | | | | | | | |
